# Evaluating the use of absolute binding free energy in the fragment optimisation process

**DOI:** 10.1038/s42004-022-00721-4

**Published:** 2022-09-05

**Authors:** Irfan Alibay, Aniket Magarkar, Daniel Seeliger, Philip Charles Biggin

**Affiliations:** 1 Department of Biochemistry, The University of Oxford, South Parks Road, OX1 3QU Oxford, UK; 2 Boehringer Ingelheim Pharma GmbH & Co. KG, Birkendorfer Str. 65, 88397 Biberach an de Riß, Germany; 3 Present Address: Exscientia Inc, Office 400E, 2125 Biscayne Blvd, Miami, FL 33137 USA

**Keywords:** Computational chemistry, Lead optimization

## Abstract

Key to the fragment optimisation process within drug design is the need to accurately capture the changes in affinity that are associated with a given set of chemical modifications. Due to the weakly binding nature of fragments, this has proven to be a challenging task, despite recent advancements in leveraging experimental and computational methods. In this work, we evaluate the use of Absolute Binding Free Energy (ABFE) calculations in guiding fragment optimisation decisions, retrospectively calculating binding free energies for 59 ligands across 4 fragment elaboration campaigns. We first demonstrate that ABFEs can be used to accurately rank fragment-sized binders with an overall Spearman’s r of 0.89 and a Kendall τ of 0.67, although often deviating from experiment in absolute free energy values with an RMSE of 2.75 kcal/mol. We then also show that in several cases, retrospective fragment optimisation decisions can be supported by the ABFE calculations. Comparing against cheaper endpoint methods, namely N_wat_-MM/GBSA, we find that ABFEs offer better ranking power and correlation metrics. Our results indicate that ABFE calculations can usefully guide fragment elaborations to maximise affinity.

## Abstract (web summary)

Fragment-based drug design is an effective approach to identifying potential binders for a given target protein, but accurately capturing changes in affinity associated with a given set of chemical modifications remains challenging. Here, the authors evaluate the use of absolute binding free energy calculations in guiding fragment optimisation decisions, finding that such calculations can usefully guide fragment elaborations to maximise affinity.

## Introduction

Over the last few decades, the fragment-based drug design (FBDD) process has matured into a popular and effective approach to designing novel binders^1^. Indeed, during the 2015–2019 period, over 131 successful fragment-to-lead campaigns were published^2–6^. With an intuitive structure-based approach, and ability to more easily sample a large chemical space, FBDD has become a strong contender to more traditional high-throughput screening methods.

In the FBDD process, a library of fragments is first screened against a given protein target in order to identify potential binders. These fragments usually adhere to the so-called “rule of three”^7^, having a molecular weight ≤ 300 Da, ClogP ≤ 3, a number of hydrogen bond donors ≤3 and a number of hydrogen bond acceptors ≤3. From this initial screen, binders are then identified through structural (e.g. X-ray crystallography, nuclear magnetic resonance (NMR)), biochemical, or biophysical (e.g. NMR, surface plasmon resonance, isothermal titration calorimetry (ITC)) characterisation^8–10^. Confirmed binders are then optimised, heavily relying on structure activity relationships to create bespoke high affinity binders. Optimising strategies either concentrate on improving the affinity of a single binder through fragment *growing*, or by combining multiple fragments occupying distinct binding sites through *linking* and *merging* decisions^11,12^.

Accurately characterising fragment-protein interactions is therefore central to the FBDD process. Despite substantial improvements in leveraging in vitro methods for fragment screening^1^, this remains a challenging task. By their nature fragments tend to be low affinity binders, somewhere in the millimolar to micromolar range, with a propensity to access multiple binding sites in a protein target. Not only do highly sensitive affinity measurements need to be employed, something that cannot always be readily used for large fragment screens, but multiple orthogonal methods are often required to validate low affinity hits^1,13^.

As a consequence, in silico methods have become increasingly popular in helping guide and support FBDD decisions^14,15^. A variety of approaches have been employed for this task, which can roughly be separated into two categories; (i) methods to identify fragment interaction sites, and (ii) methods to characterise fragment binding affinities. For the former, both simple methods such as molecular docking^16^, and more complex molecular dynamics-based approaches such as hotspot mapping^17–21^ or unbiased molecular dynamics (MD) with Markov-state modelling^22^ have shown success in identifying potential fragment binding sites on protein targets. In terms of characterising affinity, alchemical relative binding free energy (RBFE) methods have been particularly successful in ranking fragment affinities^15,23–26^. Of particular note is a 2015 study by Steinbrecher et al.^26^, which demonstrated that the FEP + RBFE tool could be used to successfully rank fragment affinities, achieving an RMSE of 1.14 kcal/mol for 96 ligands across eight fragment optimisation campaigns. Whilst RBFEs are a powerful and relatively cheap tool for this purpose, there are some disadvantages that limit its applicability in FBDD. Firstly, RBFEs do not give a direct measure of ligand binding affinities on an absolute scale, instead the method relies on other methods to normalise output free energy values. Often this would be done via an in vitro experimental measurement of a few select compounds, however as discussed above it can often be difficult to do so accurately for fragments and therefore is not always tractable in FBDD. The second major disadvantage is that RBFE protocols are generally developed to investigate small chemical perturbations on a given common chemical scaffold. This can have limited applicability in some FBDD campaigns, which will often investigate several fragments with different chemical scaffolds, sometimes even in different binding site locations. Nevertheless, we note some recent success in using RBFE methods for fragment linking purposes, although requiring several extra intermediate steps to achieve good results^27^.

As an alternative to RBFE methods, we propose that absolute binding free energies (ABFE)^28–32^ could instead be used to directly investigate fragment affinities. Despite larger computational costs, ABFEs offer a direct solution to the above described limitations of RBFEs by directly calculating the absolute free energy of binding for each individual ligand, and not requiring a transformation to another chemical entity. Previous works have shown ABFEs to offer highly accurate estimates of binding free energies across a variety of target systems, although often at increased computational costs^30,33^. Indeed, the idea of using ABFEs in fragment binding is not a novel one, and we note several other investigations employing such methods for fragment-sized molecules^15,29,34,35^. That being said, these have been mostly limited in scope and to our knowledge there have yet to be any large-scale analyses of the applicability of ABFEs to the FBDD process.

**Fig. 1:**
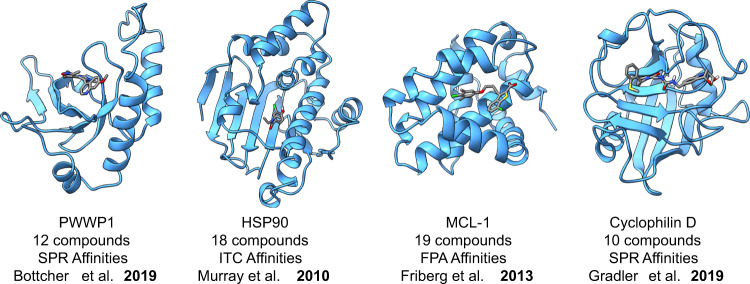
Overview of the fragment elaboration datasets. A total of 59 ligands from four different elaboration studies^38–41^ with affinities spanning the millimolar to nanomolar range are investigated here.

Here we specifically look at evaluating the use of ABFEs in the fragment optimisation process. Retrospectively calculating the binding free energies for 59 ligands across four FBDD campaigns (Fig. 1), we aim to investigate whether; (a) ABFEs offer comparable results to in vitro affinity measures, and (b) ABFEs could be used to achieve similar synthetic decisions in fragment optimisation. We also look at how ABFEs compare against cheaper methods, namely N_wat_-MM/GBSA^36,37^.

## Results and discussion

### ABFE results across all system

**Fig. 2:**
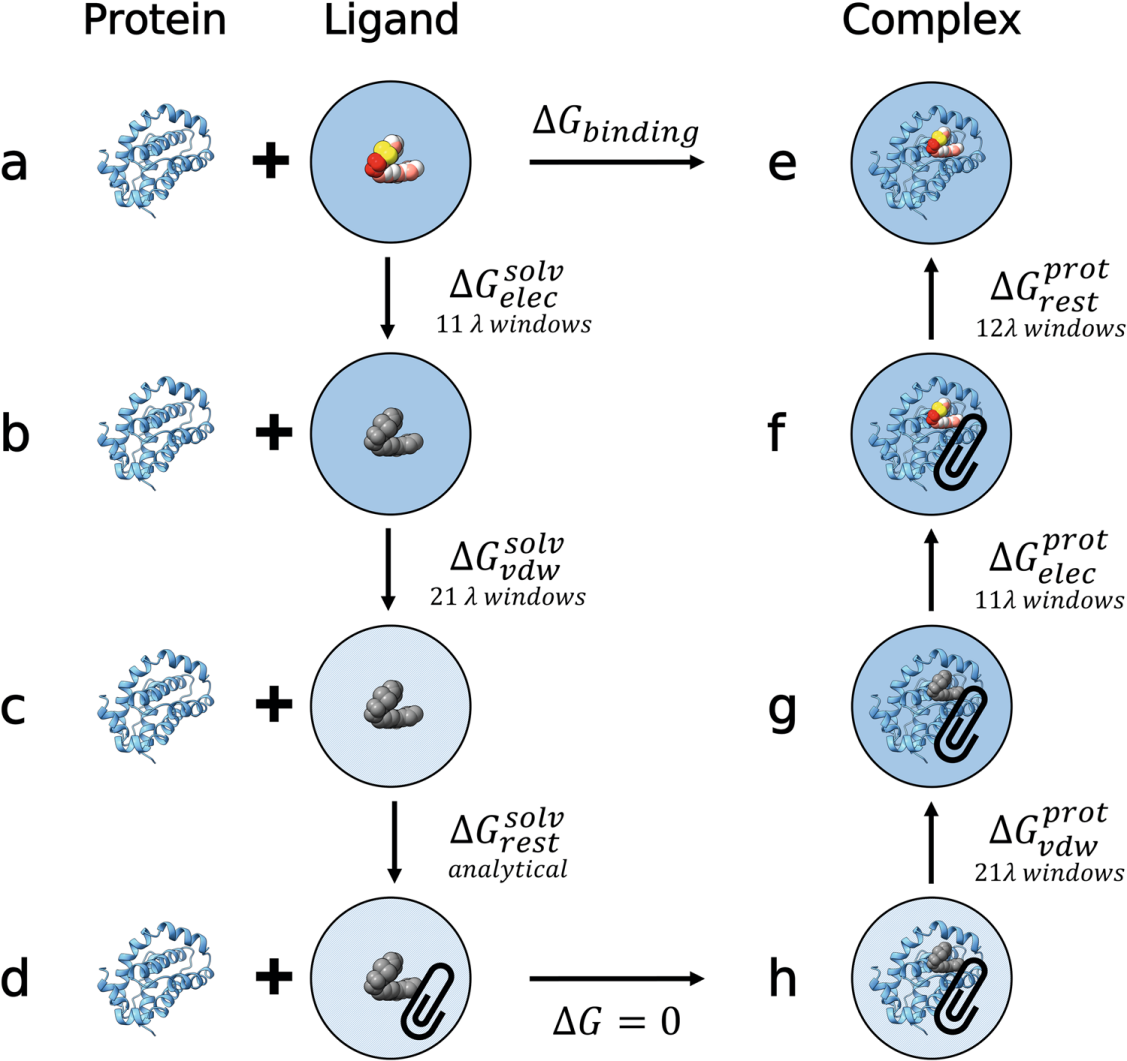
Absolute binding free energy thermodynamic cycle employed. The free energy of binding, i.e. going from a ligand in solution (state **a**) to a protein-ligand complex (state **e**), is captured through a non-physical path. First, the electrostatics are annihilated to zero (state **b**) over 11 λ windows. This is followed by a further 21 λ windows which decouple the ligand van der Waals interaction from the solvent (state **c**). The decoupled ligand is then analytically restrained as defined by ref. ^102^ (state **d**). By accounting for this restraint the ligand state is then equivalent to a non-interacting ligand in a protein-ligand complex (state **h**). The ligand interactions with the environment are then turned back on, first re-coupling the van der Waals interactions over 21 λ windows (state **g**), followed by a further 11 λ windows to add back electrostatics (state **f**). Finally, the orientational restraints are turned off over 12 λ windows resulting in a fully interacting protein-ligand complex (state **e**).

**Fig. 3:**
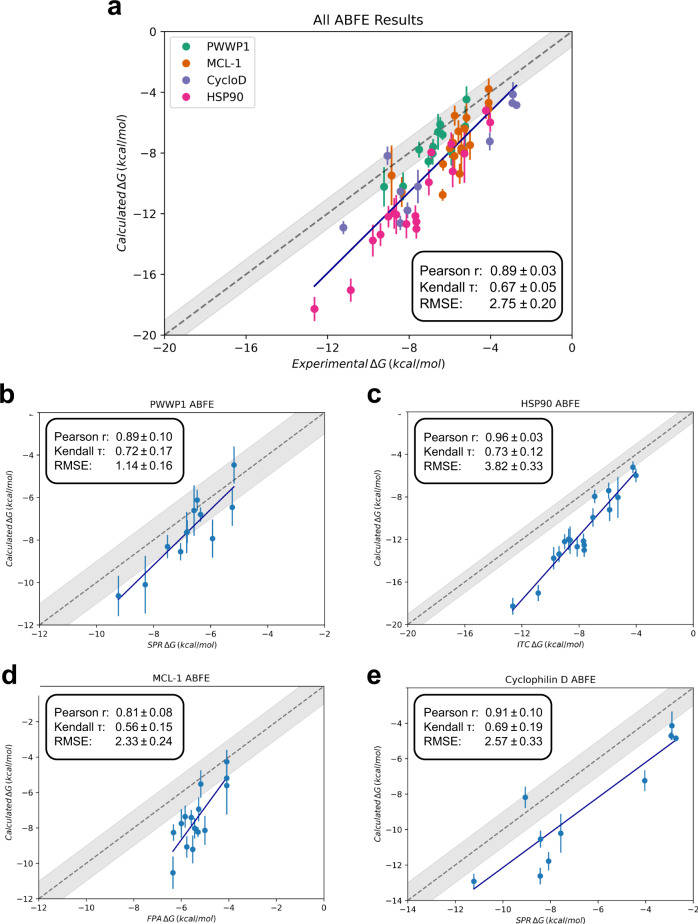
ABFE calculations results for different systems. **a** All four datasets, **b** PWWP1, **c** HSP90, **d** MCL-1, **e** Cyclophilin D. Free energy estimates are the means of the estimates across replicas, with error bars as their standard deviation. Correlation metrics calculated from the mean estimate values, with error bars derived from bootstrap resampling. All free energy results, including RMSE values, have units of kcal/mol.

The ABFE (Fig. 2) of 59 ligands (Figs. S1, S2, S4 and S5) for fragment optimisation campaigns of the PWWP1^38^, HSP90^39^, MCL-1^40^ and Cyclophilin D^41^ receptors were computed (see Methods). As shown in Fig. 3a, we see very good agreement between the calculated and experimental affinities, with a Pearson r of 0.89 ± 0.03, and a Kendall *τ* of 0.67 ± 0.05. However, we observe a RMSE of 2.75 ± 0.20 kcal/mol. As outlined in Fig. 3b–e, each dataset deviates to varying degrees from experiment, with only PWWP1 showing an RMSE close to 1 kcal/mol. The correlation shown here is on par, if not better, than other comparable alchemical free energy studies^26,42^. Whilst the RMSE is larger than the 1 kcal/mol limit shown by some other ABFE studies^32,42^, similar system dependent shifts have been reported previously^33^.

Sampling of free energies agrees well between replicas with a mean error of 0.79 ± 0.38 kcal/mol, although ~22% of the estimates show an error bar >1 kcal/mol, with the highest value being 1.93 kcal/mol. This is generally on a par with sampling errors shown by other absolute alchemical free energy studies^32,33,43^ and matching a similar level of uncertainty as the cycle closure errors shown in the Steinbrecher et al. 2015 fragment FEP + study^26^. Whilst the error in experimental measurements was not provided for all our systems, our sampling errors are for the most part approximately twice as large as the ~0.5 kcal/mol limit which we might expect from experimental measurements^44^.

### ABFEs of fragments for the PWWP1 domain of NSD3

Specifically looking at each individual case, we start with a fragment elaboration for the PWWP1 domain of NSD3 by Böttcher et al.^38^. Here 11 ligands were elaborated from an initial 160 µM fragment hit (ligand 8), eventually resulting in the 170 nM ligand BI-9321 (SI Fig. S1, Table S1). As shown in Fig. 3b, the ABFE results correlate very well with experiment, with an RMSE of 1.14 ± 0.16 kcal/mol, the lowest of all four optimisation campaigns investigated. In the original elaboration study, seven specific elaboration decisions were outlined (SI Table S2). Of these, only two would have been definitively supported by our ABFE results, that is to say—there is a greater-than-error difference between the calculated ligand binding energies in each decision. Whilst the ABFE results accurately predict the direction of the affinity change for six of the seven decisions, the majority of the changes in free energy remained within the range of the errors of the estimates. We note that the one decision where the wrong sign is predicted involves experimental ΔΔG values of −0.12 and −0.23 kcal/mol, which is well within the limit of both experimental^45,46^ and force field^47^ accuracy.

### ABFEs of a fragment optimisation for HSP90

Next, we look at a fragment elaboration study by Murray et al.^39^ elaborating 17 ligands from an initial fragment hit (ligand 3, SI Fig. S2, SI Table S4). Whilst the original study does outline two separate fragment elaboration campaigns, we specifically looked at the second elaboration set which involved a larger number of ligands. As shown in Fig. 3c, the free energy estimates correlate very well with the experimental values with a Pearson *r* of 0.96 ± 0.03 and a Kendall *τ* of 0.73 ± 0.12. However, we do see a progressive deviation from experiment as the affinity of the ligands increases leading to a RMSE of 3.82 ± 0.33 kcal/mol.

This difference between calculated and experimental values has been observed by other free energy studies of HSP90 and as detailed by Baumann et al.^48^, is possibly explained by several slow degrees of freedom associated with HSP90 binding. These include; the presence of varying waters in the binding site, ligand re-orientation, and side-chain motions. Interestingly, Baumann et al.^48^ identify that the presence of waters in the binding site worsened free energy estimates for their HSP90 test case, although it offered improved convergence in results. Whilst we have attempted to optimise the waters in the binding site using the MC/MD sampling steps in our equilibration procedure, it is possible that similar effects are impacting the accuracy of our results during the decoupling stages of our ABFE calculations. We note that in all but one ligand, ABFE calculations started with three buried waters present in the binding site (SI Fig. S3). In the case of ligand 24, for which the MC/MD procedure only added one buried water, we attempted to investigate the impact of these missing waters by calculating an ABFE with all three waters manually added to the binding site. This led to a within error change in the free energy from −12.20 ± 0.71 kcal/mol (1 water) to −11.33 ± 0.52 kcal/mol (3 waters) (SI Tables S4 and S5). From this single result, it is unclear as to how much of an influence the initial presence of waters has on the binding affinity. Future work, possibly by combining water MC steps^49^ as part of the free energy procedure (as done for RBFE in works such as those of Ben-Shalom et al.^50^ would be required to further investigate this issue).

**Fig. 4:**
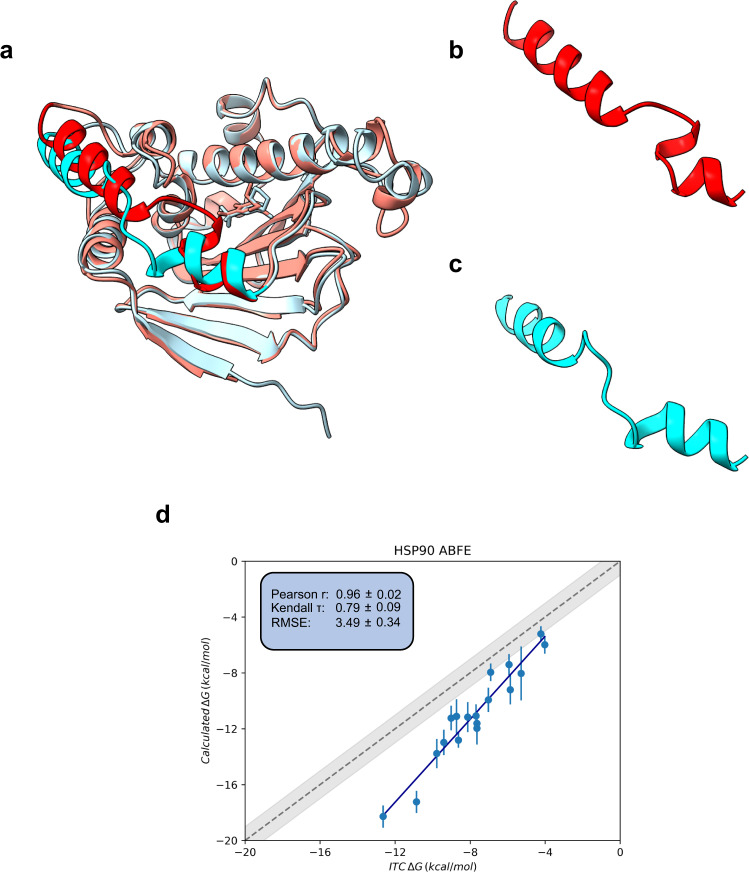
Impact of conformation on ABFE calculations for HSP90. **a** Comparison of the structures of PDB IDs 2XHT (blue) and 2XDL (red) demonstrating the difference in helicity between the two models and **b**, **c** a zoomed view of the affected helix. Residues 100–124 have been highlighted in a darker colour to aid in visualisation. PDB ID 2XAB, which is similar in structure to 2XDL and 2XHX which is similar in structure to 2XHT are not shown. **d** Absolute binding free energies of the HSP90 ligands all starting from the 2XDL-like helix loop conformation. Free energy estimates are the means of the estimates across replicas, with error bars as their standard deviation. Correlation metrics calculated from the mean estimate values, with error bars derived from bootstrap resampling. All free energy results, including RMSE values, have units of kcal/mol.

One slow motion which could have an impact here is the re-arrangement of one of the binding site loops in the region of residues ASN106 through to SER113. As shown in Fig. 4, the helicity of this loop region changes between the initial models used for ligands 3–20, 28 and 31 (PDB IDs: 2XDL and 2XAB) to the ones used for ligands 21–27, 29–30 (PDB ID: 2XHT and 2XHX). The re-arrangement of this loop from a helix-loop-helix, akin to the 2XHT conformation, to a continuous helix is well documented and previous work has shown it to be variably induced depending on the binding ligand^51–53^. In their work Murray et al. only crystalised a small subset of their ligands in either one or the other crystal forms^39^. As a result, the initial models chosen for each ligand were purely based on chemical similarity with ligands from one of the resolved structures. To investigate the impact of this loop re-arrangement on the free energies, ligands 21–27 and 29–30 were re-calculated using the 2XDL crystal as a starting conformation. As can be seen in Fig. 4d. and SI Tables S4 and S5, estimated free energies remain within error of each other despite a small 0.33 kcal/mol improvement in the mean RMSE value. Whilst not having a major impact on the results presented here, it is still possible that there are long timescale influences of this loop motion to the binding free energy which are not captured in this work. Further work using enhanced sampling methods, coupled with experimental validation of loop preference (e.g. through ATR-FTIR spectroscopy^53^) may be required to investigate the true extent of the impact of this loop motion on ABFE results.

The 2XAB and 2XDL conformations are close to those of the *apo* structure shown in PDB ID 5J2V^54^ with an all-atom alignment RMSD of 0.92 Å. We therefore do not anticipate that differences between the *apo* and *holo* structures are leading to the large overpredictions of the binding affinities seen in this fragment optimisation set, particularly those seen in the stronger binders. To verify this an ABFE of the strongest binder, ligand 31, was calculated using the 5J2V crystal as the initial protein conformer. Doing so led to a change of free energies from −18.28 ± 0.80 kcal/mol (2XAB initial structure) to −16.93 ± 0.92 kcal/mol (5J2V initial structure). Whilst indicating a subtle crystal structure dependent influence on the free energies, these two results remain within error of each other and do not account for the ~5 kcal/mol overprediction in the binding affinity compared to experiment.

In this study six elaboration decisions were outlined, of these three would have been clearly supported by our ABFE results (SI Table S6). Of the three failed decisions only one (decision 2), would have been fully miscalculated. For the other two, one involved a small change in experimental affinity (decision 5) with ΔΔG values ~0.6–0.8 kcal/mol. This likely still falls within the accuracy of ITC measurement but is too small a change to be properly captured by ABFE given the size of sampling errors. The other elaboration decision clearly identified improvements in affinity but failed to identify ligand 24 as a better binder than ligands 25 and 26 (decision 4).

### ABFEs of a fragment optimisation for MCL-1

The third system presented here is an elaboration of fragments for MCL-1 by Friberg et al^40^. In this study, two fragment series are grown in parallel, with an eventual merge to form a nanomolar compound (SI Fig S4 and Table S8). Here we have one of the lowest correlations between calculated and experimental ΔG values with a Pearson *r* of 0.81 ± 0.08 and a Kendall *τ* of 0.56 ± 0.15. We also see a shift from experiment in the absolute values, with an RMSE of 2.33 ± 0.24 kcal/mol. It should also be noted that in this series only an upper bound K_i_ of >1000 µM was assigned for three of the ligands (ligands 1, 6, and 12, SI Table S8). Disregarding these worsens correlation with experiment, yielding a Pearson *r* of 0.72 ± 0.16, Kendall *τ* of 0.43 ± 0.19, and a RMSE of 2.50 ± 0.24 kcal/mol. This rather poor correlation by comparison to the other datasets may in part be explained by the narrower activity range covered by the ligands in this series (Fig. 3d). Except from two (ligands 60 and 65), the ligands cover an ~2 kcal/mol activity range. This is possibly too narrow a range to distinguish between binding, especially given the size of the uncertainties in our calculated estimates. No apparent causes could be identified for the ~2 kcal/mol systematic shift from experiment seen in these results. Whilst no specific slow conformational changes or binding waters were identified, it is possible that unobserved long timescale motions, such as changes between *apo* and *holo* conformations, or force field inaccuracies in dealing with charged compounds may play a role here. Unfortunately to our knowledge, at the time of this work no adequate *apo* structure of MCL-1 was available for us to verify the difference in calculated absolute binding free energies between the two states.

**Fig. 5:**
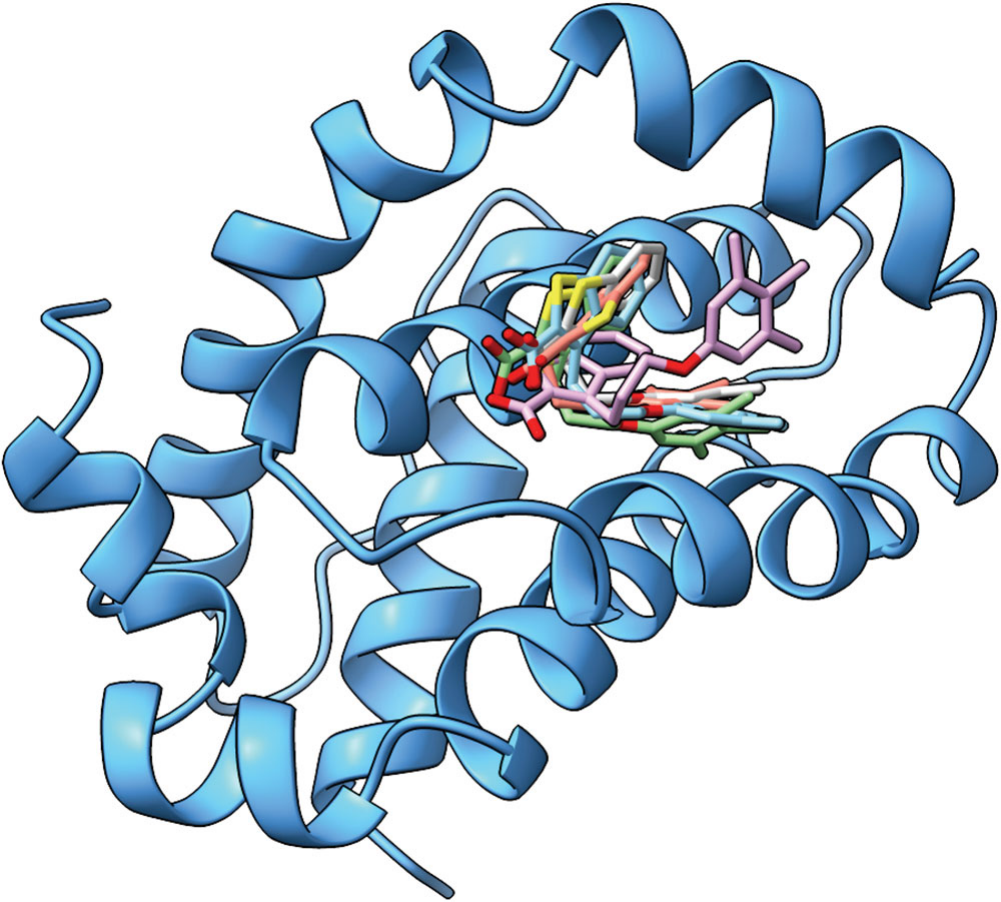
Overlay of the starting configurations of each replica of the ABFE calculations for ligand 60 in MCL-1. The re-arrangement (pink coloured ligand) of the merged ligand 60 in the MCL-1 binding site can clearly be seen.

In their paper Friberg et al.^40^ did not specifically outline a set of synthetic decisions for these ligands, but instead demonstrated four activity cliffs (SI Table S9) based on the growing of >1 mM class I fragments to micromolar ligands (decisions 1 through 3), and the merging of the class I and II ligands occupying distinct parts of the MCL-1 binding site (decision 4). The majority of fragment growing activity cliffs are clearly captured by the ABFE results, demonstrating greater than error ΔΔG values. However, both decisions 1 and 3 have one ligand pair (ligands **1**–**5**, and **3**–**13**) which are within uncertainty of each other. In addition, the predicted affinities for the optimised ligands are at times predicted to be several kcal/mol away from each other, even though they are experimentally determined to be within a ~1 kcal/mol range. The fragment merging case (decision 4) could be clearly captured by our calculated results, despite large errors in the binding free energy estimates of the merged scaffold ligands 60 and 65. Indeed, the MCL-1 dataset has on average the highest standard deviations between ABFE replicates. These large sampling errors are reflected by the charged nature of the ligands and the large conformational space accessible to the ligands within the binding site. As demonstrated for ligand 60 in Fig. 5, the ligand can effectively roll within the binding site, resulting in a pose with an RMSD > 4 Å from the others. The flexibility of binding modes for MCL-1 was also noted by Steinbrecher et al.^26^, and indicates a need for either longer simulation times or enhanced sampling schemes to reach sufficient convergence for systems such as these.

### ABFEs of a fragment optimisation for Cyclophillin D

Our final elaboration dataset is a set of fragment merging decisions for a fragment screen of Cyclophilin D by Gradler et al^41^. Here we simulate a subset of the ligands investigated in the original study, covering a total of 5 merging decisions (SI Fig. S5 and SI Table S12). We again find good correlation between the calculated and experimental free energies, with a Pearson *r* of 0.91 ± 0.10 and a Kendall *τ* 0.69 ± 0.19 albeit with a relatively large RMSE of 2.57 ± 0.33 kcal/mol. For the most part (with ligand 2 being an exception) this seems to represent a systematic shift in the free energy by ~2.5 kcal/mol (Fig. 3e). The exact cause of this shift is unclear, buried binding site waters of concern were not observed and no significant conformational differences between the *apo* and *holo* protein conformations were found. To verify the latter, the ABFE for ligand 27 was re-calculated using the *apo* crystal, PDB ID 3QYU^55^. As seen in SI Table S12, the 0.29 kcal/mol difference between the *apo* and *holo* calculated free energies sits is well within the >1 kcal/mol sampling error. Ligand conformational flexibility or force field accuracy may also play a role here, however we were unable to specifically identify the exact cause.

Of the five merging decisions captured by the simulated ligands, four would have been clearly supported by the ABFE results (SI Table S13). The large activity cliffs seen in these merging decisions, often exceeding 5 kcal/mol, are easily captured by the ABFE simulations even in cases where uncertainty exceeds 1 kcal/mol. The only miscalculated merging decision involves a relatively small ΔΔG change of 0.64 kcal/mol in experimental affinities (ligand 2 to ligand 16), which is miscalculated through ABFE by 3 kcal/mol in the opposite direction. Beyond merging decisions, whilst the estimates can differentiate between low affinity fragments (within the limit of error), the higher affinity merged ligands cannot be clearly distinguished from each other. For example, the 6 nM ligand 14, is estimated as within 0.3 kcal/mol of the 660 nM ligand 39.

### Comparison to other methods

**Fig. 6:**
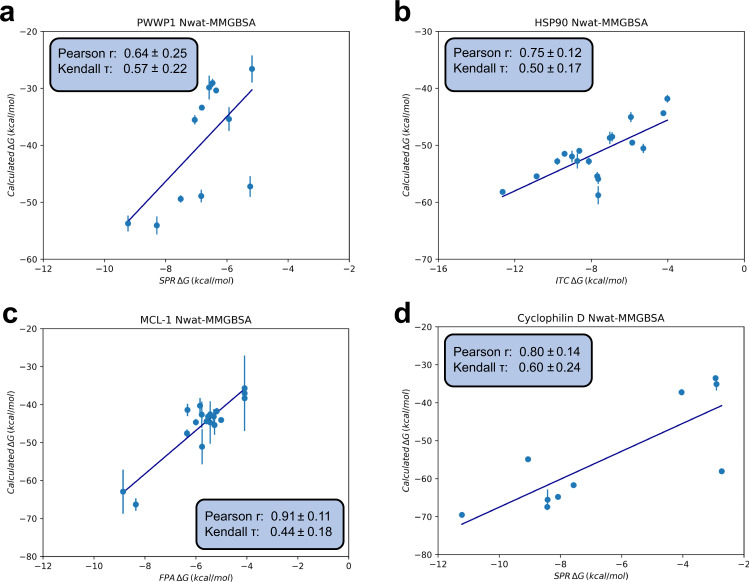
Binding free energies estimated by N_wat_-MM/GBSA. **a** PWWP1, **b** HSP90, **c** MCL-1, and **d** Cyclophilin D datasets. Free energy estimates are the means of the estimates across replicas, with error bars as their standard deviation. Correlation metrics calculated from the mean estimate values, with error bars derived from bootstrap resampling. All free energy results, have units of kcal/mol.

The computational cost of ABFEs is significant, being easily orders of magnitude higher than cheaper endpoint methods. Previous works^26,56,57^ have shown that endpoint methods such as MM/PBSA and MM/GBSA can often be competitive with alchemical methods, especially given the much-reduced computational costs. Here we compare our ABFE results with those calculated using N_wat_-MM/GBSA. As shown in Figs. 3 and 6, we find that overall ABFE calculations yield improved free energy estimates compared to N_wat_-MM/GBSA, with up to 0.2 improvements in correlation metrics (Pearson r and Kendall *τ*) for all datasets except MCL-1 where N_wat_-MM/GBSA shows a higher Pearson r of 0.91 ± 0.11. As is usual for methods like N_wat_-MM/GBSA^56^, in part as a consequence of not directly accounting for entropy^58^, the calculated absolute free energies tend to be overestimated and therefore a meaningful comparison of RMSEs between the two methods cannot be done. Analysis of the correlation of signed errors between both methods (Supplementary Note S4 and SI Fig. S8) shows that for PWWP1 and HSP90 the same ligands seem to lead to largest deviations from experiment. This could indicate that for these systems errors may predominantly stem from inaccuracies in the model (e.g. force field) rather than purely sampling errors. For the other systems, Cyclophilin D and MCL-1, no such trend is observed. However, we also note that an analysis of potential outliers (Supplementary Note S5 and SI Figs. S10–S12) does not show much overlap in identified outliers between the two methods, with more outliers identified for N_wat_-MM/GBSA, in part due to a wider range in predicted free energy values.

Despite a lower predictive performance in ranking binders, the N_wat_-MM/GBSA method shows a reasonably good accuracy in ranking fragment optimisations, being able to clearly predict several of the elaboration decisions (SI Tables S3, S7, S10, S14). Surprisingly, a similar amount of decisions to the ABFE calculations are predicted here. Whilst the N_wat_-MM/GBSA results predict one fewer decision for the HSP90 dataset, two more decisions are predicted for PWWP1. In addition, for MCL-1 whilst the fragment optimisation activity cliffs for decision 1 cannot be clearly distinguished due to large uncertainties, the optimisation of ligand 12 into ligand 13 (decision 3), which could not be supported by the ABFE results can be clearly identified using N_wat_-MM/GBSA.

**Fig. 7:**
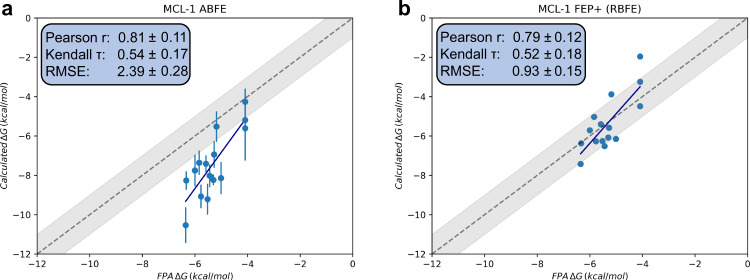
Comparison of a subset of the MCL-1 dataset. **a** ABFE simulations and **b** the 2015 FEP + study by ref. ^26^ Free energy estimates are the means of the estimates across replicas, with error bars as their standard deviation. Correlation metrics calculated from the mean estimate values, with error bars derived from bootstrap resampling. All free energy results, including RMSE values, have units of kcal/mol.

We can also compare some of the results with previously published RBFE data. Specifically, a subset of the MCL-1 dataset investigated here was also calculated using FEP + in Steinbrecher et al.’s 2015 fragment optimisation study^26^. Whilst only offering a limited comparison, we find the two methods to give comparable results (Fig. 7), with the two methods offering within error values for Pearson r and Kendall *τ*. Looking at the signed errors from experiment (SI Fig. S9), there appears to be correlation between the two methods indicating that similar ligands deviate the most from experiment. This concurs with the observation from the N_wat_-MM/GBSA results that indicate that errors in the model (e.g. force field) may have a stronger influence in the MCL-1 set than sampling errors.

Comparison of retrospective elaboration decisions (SI Tables S9 and S11) shows that FEP + is also able to accurately predict the correct changes in free energies for the elaborations in this subset of MCL-1. Although an analysis of errors cannot be made as errors between repeats were not provided in the Steinbrecher et al. study^26^ the relatively low cycle closure errors for these ligands (for the most part lower than 1 kcal/mol) indicate that FEP + would likely outperform the ABFE results shown here (with errors exceeding 1 kcal/mol) in clearly identifying these elaborations. Given the narrow activity range and the relatively large uncertainties in the estimates, further comparison of correlation between calculated and experimental values is unlikely to yield much insight. We do note that the reported RMSE for FEP + is much lower at 0.93 kcal/mol, however the absolute numbers in this FEP + study were normalised based on the experimental affinity and therefore cannot be directly compared to the ABFE absolute results which required no a priori experimental affinity data.

## Conclusions

In this study, we demonstrate that absolute binding free energy calculations can successfully estimate affinity changes in the fragment optimisation process. Whilst not always being able to clearly support elaboration decisions, the right direction in the affinity change was captured for the vast majority of cases (46 of the 52 ligand pairs involved in the 22 elaboration decisions). Despite occasional challenges in accurately matching experimentally derived binding free energies, the predictive power displayed here shows that ABFEs can be suitably used to not only act to orthogonally validate fragment hits but also to guide fragment optimisation.

We also show that ABFE calculations outcompete cheaper endpoint methods such as N_wat_-MM/GBSA. Nevertheless, the N_wat_-MM/GBSA method employed here showed reasonably good predictive ability, particularly in supporting retrospective elaboration decisions, and could easily be used as a low computational cost prefilter to more expensive ABFE calculations. The N_wat_-MM/GBSA method employed here is also a very simplistic one, accounting directly for entropy^59–61^ and using methods employing independent trajectories for the complex, protein and ligand components may yield improved results. For one system in our benchmark set, MCL-1, we also found that ABFEs offer comparable results to RBFE methods Given the lower computational costs of relative binding free energies, the decision as to whether or not they should be used preferentially to absolute calculations is likely to depend on the use case. In cases where one attempts to elaborate from a common core structure, RBFEs are likely to offer better convergence at much cheaper compute requirements. However, when dealing with issues such as scaffold hopping and fragment linking (as shown in the Cyclophilin D set), although we note recent success in employing FEP + for such cases^27^, ABFEs become a lot simpler, particularly in the number of intermediate steps involved. The comparison between the two methods shown here is too limited to offer a complete evaluation of the differences in efficiency between RBFEs and ABFEs in fragment optimisation, particularly in fragment linking cases. Such an evaluation may form the basis of future work in this area. It is also worth mentioning that whilst ABFEs do have a much larger computational cost compared to end-point or RBFE methods, advances in GPU utilisation are making the high-throughput use of such a technique more tractable.

Our results demonstrated cases of both precision and accuracy limitations. This was either seen through poor inter-replica convergence (as shown in some of the MCL-1 ligands), or large deviations from experimental affinities (as shown in HSP90). The exact causes of some of the large deviations seen in these benchmarks, particularly for HSP90 and Cyclophilin D, could not be identified. Whilst still likely playing a role, the influence of bound waters and *apo-to-holo* protein conformational changes did not appear to be main contributors to these free energy deviations. One potential cause could be inadequate sampling of slow processes not easily identifiable from the short 20 ns simulations employed here. Indeed, we note that the ABFE protocol used here is reasonably simple and could easily be improved to address some of these sampling limitations^62^. This could not only improve the precision of our results, but also possibly the accuracy by more readily capturing rare events occurring during the ligand binding process. For example, recent work by Khalak et al.^30^ demonstrates that non-equilibrium absolute binding free energy can be used to bridge differences between large conformational differences between bound and unbound states. The use of higher quality force fields, especially via bespoke ligand parameterisations^63,64^, may also be key to alleviating some of the accuracy limitations identified here. We hope that future work will focus on improving both the precision and accuracy of ABFE results for fragment optimisation.

It is important to also note that a priori system specific knowledge remains crucial in ensuring the performance of alchemical methods. Whilst one of the main advantages of ABFE calculations is the direct estimate of binding affinity, having access to experimental affinities for a subset of the calculated binders to the same target or a structurally similar one, would significantly help in identifying and adjusting issues in a model. For example, we were able to confidently use an N-terminal truncated model of the PWWP1 system (see Supplementary Note 2) after observing that initial calculated free energies were close to those obtained from experiment. Similarly, the overestimations in the free energy estimates seen here are sufficiently large that they could mislead synthetic efforts, but also could easily be adjusted through prior affinity data on a few of the data points.

Despite the positive results displayed here, it is important to make clear that in many ways this benchmark only tackles a simplified set of tasks in the FBDD process and that several challenges remain ahead in validating and using ABFE. Of particular note, this dataset solely looked at cases where most binding poses were known and as a consequence were likely to not include ligands occupying more than one binding pose significantly contributing to the total free energy. Thus, our use of orientational restraints in this study could be justified. Whilst one could envision using ABFEs to identify optimal poses, an initial assessment (Supplementary Note S6, Table S16) indicate that this is unlikely to be a simple task. Indeed there are FBDD cases where accounting for multiple binding poses either through less restrictive restraints, such as center of mass restraints^48,65–68^ or through enhanced sampling means (e.g. NCMC^69^ or metadynamics^70^), or by explicitly accounting for multiple known poses^71,72^ will be required. In addition, as mentioned in our methods we also specifically attempted to avoid known cases where issues may arise in ABFE calculations, such as the presence of structural ions, membrane proteins, or ambiguous protonation states. Future work will look at tackling some of these more complex issues, particularly within the context of FBDD and see what type of solutions can be leveraged to ensure the accurate calculation of ABFE. There is still a lot of work to be done if these methods are to be employed routinely in design decisions in future, although progress is encouraging and not just in simple systems^73^.

## Methods

### System selection

Four previously published FBDD campaigns^38–41^ (Fig. 1), each with a different protein target, were selected for this benchmark. The systems were selected based on the availability of high-quality experimental measures, with the presence of both crystallographic and affinity data. We also looked to cover a wide activity range, spanning from millimolar to nanomolar affinities, and various different fragment optimisation cases, such as the presence of multiple distinct binding sites, changes in net charge, fragment growing and fragment merging (but no fragment linking cases). Note that some cases were specifically avoided to ensure this study remained tractable. For example, large multimeric protein targets were not included to reduce computational costs. Similarly, membrane proteins and systems with binding site metals or known protonation issues were not chosen to avoid introducing additional complexity in this initial dataset. Ideally, the experimental measurements across all four datasets would be the same (e.g. all ITC measurements of affinity) as this is potentially a source of systematic error. This unfortunately was not the case here (Fig. 1). Some of the pitfalls associated with experimental equilibrium binding measurements have recently been highlighted^74^.

### System preparation and simulation details

As structural data was not available for all protein-ligand complexes, the initial configurations were generated by modifying a chemically close protein-ligand crystal structure using open source PyMOL^75^ (see Supplementary Note S1 for full details). If alternate states were available in the starting crystal, state A was always retained. Where appropriate, acetyl and N-methyl caps were also added to protein structures using PyMOL. Protons were assigned using protoss^76,77^, as made available within the ProteinPlus web server^78,79^. In the case of the PWWP1 domain, missing loop residues were modelled using modeller 9v21^80^ and the DOPE-HR scoring method. Of the 500 generated models, the top 10 models were rescored using the QMEAN^81^ scoring function as implemented in SWISS-MODEL^82^, with the final model selected as the best QMEAN-scored model. The PWWP1 N-termini was also truncated at isoleucine 393 to avoid long timescale interactions between residues in the disordered N-termini region and the ligand binding site (see Supplementary Note S2 and SI Fig. S6).

Solvation and force field assignment was achieved through a combination of AmberTools 18^83^ and GROMACS 2019^84^. Ligand parameters and partial charges were assigned using the GAFF2 force field and the AM1-BCC partial charge model. The ff99SB-ILDN force field was used for protein parameters and the complexes were solvated in TIP3P^85^ cubic boxes with a minimum distance of 12 Å from the solute to the box edge using GROMACS’ *solvate* module. Sodium and chloride ions were added to neutralise the systems and achieve a concentration of 150 mM using the *genion* module of GROMACS. ParmEd version 3.2.0 (https://github.com/ParmEd/ParmEd) was used to convert input topologies and coordinates between AMBER and GROMACS file formats.

Unless otherwise mentioned, a hydrogen mass repartitioning scheme (HMR)^86,87^ was used to achieve a 4 fs integration timestep for our simulations. Due to differences in how MD engines handle hydrogens with analytical constraints^87^, hydrogen masses, except those of waters, were increased to 3 and 4 atomic mass units for AMBER and GROMACS respectively. Whilst HMR has been employed before in alchemical free energy calculations^88–90^, there is limited data on its use in ABFE in GROMACS. To this end, a small validation using the Cyclophilin D dataset is shown in Supplementary Information (see Supplementary Note S3, SI Fig. 7 and SI Table S15). Water hydrogen motions were constrained using SETTLE^91^ and either SHAKE^92^ or LINCS^93,94^ for other constraints in AMBER or GROMACS respectively. In all cases, simulation temperature was maintained at 298.15 K though Langevin dynamics with a collision frequency of 2 ps^−1^. A simulation pressure of 1 atmosphere was maintained using various barostats. For AMBER simulations, a Monte Carlo barostat^95^ was employed with a volume exchange attempt frequency of 100 ps. For GROMACS, the initial equilibration steps used the Berendsen barostat^96^ with a time constant of 1 ps, followed by the Parrinello-Rahman barostat^97^ with a time constant of 2.0 ps for all follow-on equilibration and production simulations. In all cases, a cut-off of 1 nm was used for short range interactions, and long range electrostatics are handled via PME^98,99^. Input topologies, coordinates, and simulation control files are provided as Supplementary Information (zenodo: 10.5281/zenodo.5913469 and https://github.com/bigginlab/fragment-opt-abfe-benchmark).

### System equilibration

A two-step equilibration procedure is followed here. First, binding site waters were equilibrated using the AMBER MC/MD procedure^49^ as implemented in AMBER18’s pmemd.cuda engine^100,101^. The system first underwent a short initial equilibration phase consisting of 10,000 steps of minimisation, followed by a 500 ps NVT phase, and then 5 ns of NPT. During this equilibration, protein backbone atoms and non-hydrogen ligand atoms were restrained using a 5 kcal/mol/Å^2^ force constant. The system then underwent a 5 ns MC/MD with the same positional restraints with 25 000 MC attempts every 1000 MD steps. This was followed by a further 10 ns of MC/MD water exchange with position restraints on the ligand removed. In all MC/MD simulations, the NVT ensemble was sampled and the MC swap region box was trimmed to ensure as many exchanges with binding site waters as possible.

Once complete, the final frame from the MC/MD procedure was converted to GROMACS using ParmEd. The system was then further equilibrated using the *mdrun* engine in GROMACS 2019^84^. This included an initial 10,000 step minimisation, followed by 1 ns of restrained (2.39 kcal/mol/Å^2^ applied to the protein backbone and ligand non-hydrogen atoms) NVT and NPT equilibration. The latter step employed the Berendsen barostat^96^ as detailed above. The system was then relaxed using 5 ns of unrestrained NPT simulation using the Parrinello-Rahman barostat^97^. Finally, a further 20 ns of NPT simulation was generated. This final 20 ns simulation was used to both derive Boresch-style^102^ orientational restraint parameters and for N_wat_-MM/GBSA analysis.

### Absolute binding free energy calculations

Here we employed an ABFE protocol similar to the one previously described by Aldeghi et al.^31,32^. Following the equilibration phase, a partial decoupling scheme is employed to trace the alchemical path from a fully interacting protein-ligand complex to a ligand in solution as shown in Fig. 2. This partial decoupling scheme involves annihilating ligand partial charges through 11 windows spaced at λ intervals of 0.1 from each other. A charge annihilation scheme was used here in order to avoid known issues with nonbonded exclusions when using the free energy code in GROMACS 2021 (see https://manual.gromacs.org/2021-current/). The charge decoupling is then followed by 21 Van der Waals decoupling windows spaced with the following λ schedule [0.0, 0.05, 0.1, 0.15, 0.2, 0.25, 0.3, 0.35, 0.4, 0.45, 0.5, 0.55, 0.6, 0.65, 0.7, 0.75, 0.8, 0.85, 0.9, 0.95, 1.0]. A soft-core potential for decoupled Van der Waals interactions was used^103^. In addition, to restrict ligand motion in the complex phase an orientational restraint, as defined by Boresch et al.^102^, was employed. This restraint was applied over 12 windows in the complex decoupling phase with the following schedule [0.0 0.01, 0.025, 0.05, 0.075, 0.1, 0.15, 0.2, 0.35, 0.5, 0.75, 1.0]. In the solvent phase, the influence of this restraint was accounted for analytically.

Appropriately choosing which 6 atoms to involve in Boresch-style^102^ orientational restraints can be a complex issue, here we use the procedure as implemented in MDRestraintsGenerator (10.5281/zenodo.4570555). Briefly, we pick out the least mobile ligand atoms from our final 20 ns equilibration simulation (see *System Equilibration* above) as potential anchor points for our orientational restraint. We then analyse the trajectory to select all available alpha carbon protein anchor atoms within an 8 Å cut-off of the ligand anchor atoms, generating a list of potential orientational restraints (where the nearest bonded ligand heavy atoms and protein backbone atoms are selected as the remaining atoms involved in the orientational restraint). Bond, angle and dihedral timeseries for all identified restraints are obtained and the restraint with the lowest standard deviation across all values is picked as our orientational restraint of choice. The frame closest to the mean bond, angle and dihedral values of the restraint over the 20 ns simulation is then used as the starting point for the ABFE cycle. With each replica undergoing independent equilibrations, although starting from the same initial structure, we obtain a different restrained conformation for each replica, helping us better capture the impact of conformational flexibility on the calculated free energy.

Each ABFE window consists of a short equilibration similar in protocol to the previously detailed equilibration cycles, although using 10 ps for the restrained NVT equilibration, 100 ps for the restrained NPT Berendsen barostat equilibration, and 500 ps for the unrestrained NPT Parinnello-Rahman barostat equilibration. This is followed by 20 ns of production NPT simulation. A total of five independent replicas of the ABFE cycle are simulated. For the protein-ligand complexes, each replica is independently equilibrated and uses different orientational restraints, allowing the replicas to more representatively sample the available conformational space within the binding site. GROMACS 2021 was used for all ABFE calculations. For charged ligands, an analytical correction was used to account for finite size errors as detailed by Rocklin et al.^104^. To achieve this, the ABFE simulations were carried out with a net system charge in the fully coupled state by adding or removing a counterion as necessary. Files containing the sampled ΔH and ΔH/Δλ energies have been made publicly available at zenodo: https://zenodo.org/record/5906262, https://zenodo.org/record/5906110, https://zenodo.org/record/5904110, https://zenodo.org/record/5906019 and https://zenodo.org/record/5906805)

### N_wat_-MM/GBSA calculations

The last 20 ns of our equilibration procedure was analysed using the N_wat_-MM/GBSA method^36,37^. Here the *N* = 20 nearest waters to the ligand in the binding site are included as part of the protein in the MM/GBSA calculation. As is typical for N_wat_-MM/GBSA calculations, a single trajectory approach was employed, and no entropy corrections were included. The N_wat_-MM/GBSA method was chosen here due to its simplicity, low computational costs, and previous work demonstrating N_wat_-MM/PBSA to have good accuracy in ranking Bromodomain-binding ligands compared ABFE calculations^56^. GROMACS XTC files were converted to AMBER NETCDF format using MDAnalysis 1.1.1^105,106^, and the trajectory and topologies manipulated to remove excess waters using cpptraj v5.1.0^107^. The mmbondi2 radii with GB model 2 parameters^108^ were employed with a GB salt concentration of 150 mM. Frames were sampled every 25 ps from the 20 ns trajectories. The AmberTools21 versions of MMPBSA.py and *sander* were used for the MM/GBSA calculations^109^.

### Analysis

Analysis of the alchemical simulations was achieved using the alchemlyb v0.3.0 library (https://zenodo.org/record/3361016) ^110^ and the MBAR estimator as implemented in pyMBAR v3.0.3^111^. The first 1 ns of each production window was discarded as extra equilibration time and samples were decorrelated based on the derivative of the potential with respect to λ up to a maximum frequency of 100 ps^−1^. Sampling error is shown as the standard deviation of the mean free energy estimates across all five repeats. Where appropriate, an analytical correction was included to account for finite size errors as defined by Rocklin et al.^104^ and implemented in rocklinc (https://github.com/xiki-tempula/rocklinc), using ABPS v3.0^112,113^ for Poisson-Botlzmann calculations^113^. It should be noted that no additional long range dispersion corrections (e.g. EXP-LR)^114^ were employed here.

All other simulation analyses were carried out using MDAnalysis 1.1.1^105,106^, spyrmsd for symmetry corrected RMSDs^115^, and the numpy^116^, scipy^117^, and scikit-learn^118^ libraries. Plotting was done using the matplotlib library^119^, and images of atomic coordinates through ChimeraX^120^. The correlation between the calculated and experimental affinities are analysed via Spearman *r*, Kendall *τ*, and RMSE, with error bars obtained as the standard deviation of the means generated through bootstrap resampling (100,000 iterations).

### Evaluation of synthetic decisions

As part of our analysis we attempt to retrospectively evaluate the influence our predicted values would have had on the original synthetic decisions undertaken in the fragment optimisations. Overall this is a rather difficult task with many caveats, including; synthetic decisions not always being well defined, steps involving testing affinity changes between more than one ligand pair, and inequalities in the difficulty between synthetic steps. For example; for PWWP1 Böttcher et al.^38^ first detail investigating the impact of adding a 3,5-dimethyl-1,2-oxazole moiety on ligand 8 into order to form ligand 9. The authors then detail their next step as testing 2-methyl- and 2,6-dimethyl-phenyl substituents instead of the dimethyl-1,2-oxazole, showing moderately improve potency (ligands 10 and 11). Given the context of the tested substituents, one could look at these as either two or three optimisation decisions, based on whether one considers the synthesis of ligands 10 and 11 as a combined test of affinity change in the optimisation process. Here we have, to the best of our ability, attempted to group these syntheses into decisions based on the text outlined in the source reference (see SI Tables S2–3, S6–S7, S9–S11, and S13–14. This was easily done for HSP90^39^ and PWWP1^38^ but was more complex for MCL-1^40^ and Cyclophilin D^41^. For MCL-1 we opted to group the decisions into three sets of substitutions, each for a specific fragment core, and one fragment merging decision. For Cyclophilin D, five clear fragment merging cases could be identified from the simulated ligands and these were used as the synthetic decisions. One ligand from the PWWP1 set, ligand 14, was not included in the elaboration decisions. This is due to the main text rationalising the synthesis of this ligand based on the affinity of a ligand which was not included in our calculated set due to its large size (identified as ligand 7 by Böttcher et al.^38^). We nevertheless kept ligand 14 in our calculated set as it was structurally similar to the other PWWP1 ligands and offers a convenient extra data point towards evaluating the accuracy of ABFE calculations relative to experiment. It is also worth noting that the non-equal number of syntheses per decision, combined with our assessment that a failed decision occurs when any one ΔΔG difference in that set is insufficiently estimated, makes it such that some decisions have a much higher predictive difficulty than others.

## Supplementary information

Biggin_PR File

Supplementary Information

## Data Availability

Input topologies, coordinates, and simulation control files are provided at https://zenodo.org/record/5913469 and https://github.com/bigginlab/fragment-opt-abfe-benchmark. The implementation of the MDRestraintsGenerator can be found here: https://zenodo.org/record/4570556 Files containing the sampled ΔH and ΔH/Δλ energies have been made publicly available at zenodo: https://zenodo.org/record/5906262, https://zenodo.org/record/5906110, https://zenodo.org/record/5904110, https://zenodo.org/record/5906019 and https://zenodo.org/record/5906805.
