# Supplementary material for: Evaluating the use of absolute binding free energy in the fragment optimisation process
Source: Commun Chem. 2022 Sep 5;5:105. doi: 10.1038/s42004-022-00721-4 (PMC9814858; doi:10.1038/s42004-022-00721-4)
Supplement: Supplementary file 2 — Supplementary Information [file 42004_2022_721_MOESM2_ESM.pdf]

Supplementary Information

Evaluating the use of Absolute Binding Free Energy in the fragment optimization process

Irfan Alibay[a], Aniket Magarkar[b], Daniel Seeliger[b,c], Philip Charles Biggin[a],

[a]The University of Oxford, Department of Biochemistry, South Parks Road, Oxford, UK, OX1 3QU

[b]Boehringer Ingelheim Pharma GmbH & Co. KG, Birkendorfer Str. 65, 88397 Biberach an de Riß, Germany

[c]Present address: Exscientia Inc, Office 400E, 2125 Biscayne Blvd, Miami, FL 33137. USA

*Correspondence to: philip.biggin@bioch.ox.ac.uk

Supplementary Note S1. Dataset details

In this study, four fragment elaboration studies are retrospectively analysed. These target; PWWP1[1], HSP90[2], MCL-1[3], and Cyclophilin D[4]. Here we provide further details on the various systems investigated including; ligand structures, template protein-ligand complex for input generation, experimental and calculated free energies, and elaboration decisions. To facilitate comparison with the original studies, the ligand identifications from the source paper are used here.

The aforementioned template protein-ligand complex refers to the initial PDB structure used to generate the relevant protein-ligand complex. Using open source PyMOL[5], ligand structures were modified from this initial template, and where necessary side-chains in the binding site were rotated to avoid clashes. All input structure files are made available separately.

Experimental free energies are derived from either K_D or K_i values, and are converted to Gibbs free energies through the expression $\Delta G = RT \ln K_D$. In all cases a temperature of 298.15 K is used. In all but the MCL-1 dataset this is specifically mentioned as the experimental temperature. For MCL-1, the temperature is defined as “room temperature”, and has therefore been assumed to also be 298.15 K.

The identified elaboration decisions were based on the text of the original source materials. Where possible, sets of elaborations are grouped as decision steps based on the apparent intent of the chemical modifications. A decision step is identified as being successfully predicted by a method if both the right direction in the affinity is achieved and the change in free energy ($\Delta\Delta G_{calc}$) is greater than the uncertainties in both the starting and final ligand free energy estimates (labelled as Sum(Error)). In the sole case where no improvement is expected, decision 1 of the HSP90 dataset, then a successful decision is decided as a $\Delta\Delta G_{calc}$ which is less than the sum of the uncertainty in the estimates. For the MCL-1 dataset, an analysis of retrospective elaboration decisions was also done for FEP+ results as obtained from Steinbrecher et al.’s fragment benchmark study[6]. In this specific case the Sum(Error) is not reported as error bars for the derived absolute free energies were not provided in the original study. Nevertheless, the relatively low cycle closure errors shown in Steinbrecher et al’s work indicates that the elaborations would likely be greater than error.

1.1 PWWP1

For the PWWP1 domain of NSD3, we explore a series of 12 ligands demonstrating the optimisation of an initial 160 μM fragment structure (ligand 8) through to a 170 nM ligand (BI-9321) [1]. For this dataset, an initial X-ray structure of ligand 8 binding to PWWP1 (PDB ID: 6G2B) was used as the template protein-ligand complex for all the ligands.

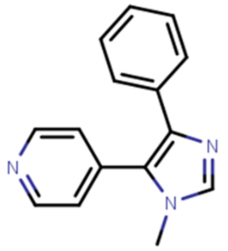

ligand 8

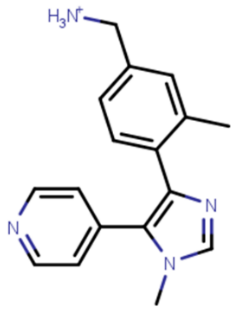

ligand 12

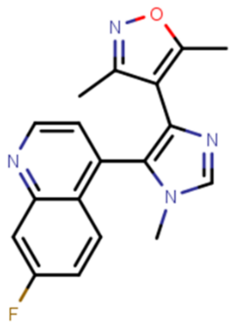

ligand 16

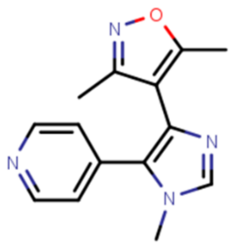

ligand 9

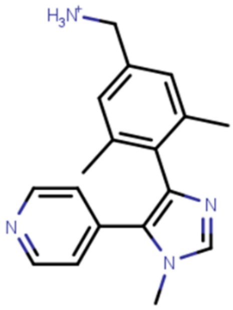

ligand 13

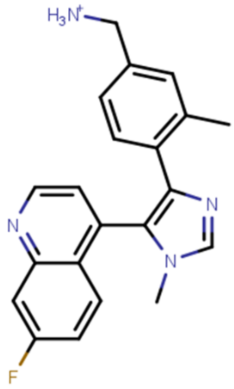

ligand 17

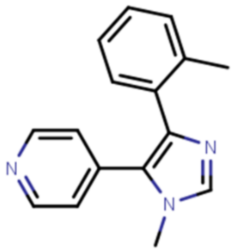

ligand 10

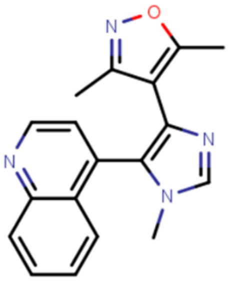

ligand 14

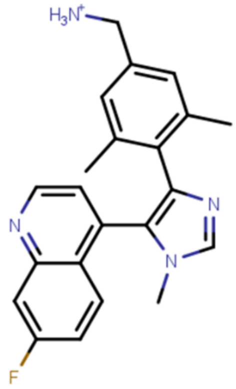

ligand BI-9321

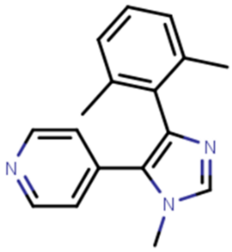

ligand 11

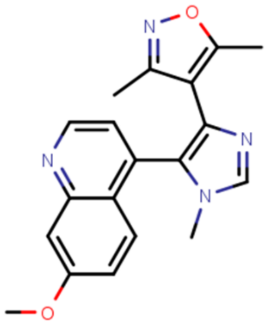

ligand 15

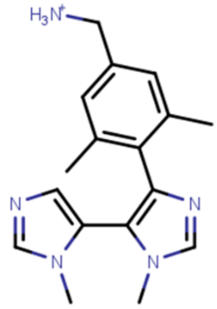

ligand BI-9466

***Figure S1**. Structures of the 12 PWWP1 ligands*

Table S1. *Experimental and calculated binding free energies for the PWWP1 ligands. ΔG^{ABFE} and ΔG^{MMGBSA} refers to the calculated absolute binding free energy (ABFE) and N_{wat}-MM/GBSA values respectively. All energies in kcal/mol.*

Ligand ID	ΔG^{exp}	ΔG^{ABFE}	$\Delta G^{MM/GBSA}$
8	-5.18	-4.47 ± 0.87	-26.60 ± 2.38
9	-6.35	-6.80 ± 0.34	-30.35 ± 0.52
10	-6.47	-6.12 ± 0.49	-29.06 ± 0.72
11	-6.58	-6.61 ± 1.19	-29.86 ± 2.10
12	-7.51	-8.31 ± 0.55	-49.42 ± 0.62
13	-6.84	-7.65 ± 0.97	-48.91 ± 1.11
14	-6.82	-7.61 ± 0.54	-33.39 ± 0.36
15	-5.93	-7.93 ± 0.90	-35.39 ± 2.09
16	-7.05	-8.55 ± 0.41	-35.53 ± 0.82
17	-8.29	-10.10 ± 1.36	-54.06 ± 1.58
BI-9321	-9.23	-10.63 ± 0.95	-53.72 ± 1.40
BI-9466	-5.24	-6.46 ± 0.87	-47.23 ± 1.84

Table S2. *Evaluation of the ligand elaboration steps of the PWWP1 ligand set for the ABFE results. All energies in kcal/mol.*

Decision	Starting ligand ID(s)	Final ligand ID(s)	Elaboration outcome						
			ΔΔGexp	ΔΔGcalc	Sum(Error)	Expected outcome	ΔΔGcalc > Sum(Error)	Right direction?	Clear decision?
1	8	9	-1.17	-2.33	1.21	Improved affinity	Yes	Yes	Yes
2	9	10	-0.12	0.68	0.83	Improved affinity	No	No	No
		11	-0.23	0.19	1.53		No	No	
3	10	12	-1.04	-2.19	1.04	Improved affinity	Yes	Yes	No
	11	13	-0.26	-1.04	2.16		No	Yes	
4	13	9466	1.60	1.19	1.84	Reduced affinity	No	Yes	No
5	15	16	-1.12	-0.62	1.31	Improved affinity	No	Yes	No
6	12	17	-0.78	-2.32	1.88	Improved affinity	Yes	Yes	No
	16		-1.24	-1.55	1.77		No	Yes	
7	13	9321	-2.39	-2.59	1.68	Improved affinity	Yes	Yes	Yes
	16		-2.18	-2.08	1.36		Yes	Yes	

Table S3. *Evaluation of the ligand elaboration steps of the PWWP1 ligand set for the N_{wat}-MM/GBSA results. All energies in kcal/mol.*

Decision	Starting ligand ID(s)	Final ligand ID(s)	Elaboration outcome						
			ΔΔGexp	ΔΔGcalc	Sum(Error)	Expected outcome	ΔΔGcalc > Sum(Error)	Right direction?	Clear decision?
1	8	9	-1.17	-3.75	2.90	Improved affinity	Yes	Yes	Yes
2	9	10	-0.12	1.29	1.24	Improved affinity	No	No	No
		11	-0.23	0.49	2.62		No	No	
3	10	12	-1.04	-20.36	1.34	Improved affinity	Yes	Yes	Yes
	11	13	-0.26	-19.05	3.20		Yes	Yes	
4	13	9466	1.60	1.68	2.95	Reduced affinity	No	Yes	No
5	15	16	-1.12	-0.14	2.91	Improved affinity	No	Yes	No
6	12	17	-0.78	-4.64	1.44	Improved affinity	Yes	Yes	Yes
	16		-1.24	-18.53	1.33		Yes	Yes	
7	13	9321	-2.39	-4.30	2.02	Improved affinity	Yes	Yes	Yes
	16		-2.18	-18.19	2.22		Yes	Yes	

1.2 HSP90

In their original study, Murray *et al.*[2] outline two sets of fragment optimisations for HSP90. Here we specifically look at the second optimisation set starting from the 790 μM ligand 3 through to the 540 pM ligand 31. Four different template protein-ligand complexes were used to generate the initial complexes. These are PDB IDs 2XDL (ligands 3-20), 2XHT (ligands 21-23, 25-27, 29-30), 2XHX (ligand 24) and 2XAB (ligands 28 and 31). Further ABFE calculations using different starting structures are shown in Table S5.

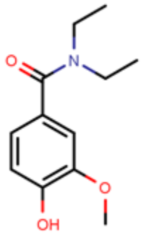

ligand 3

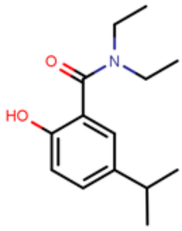

ligand 19

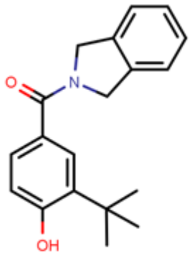

ligand 24

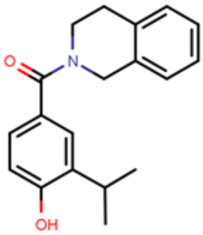

ligand 29

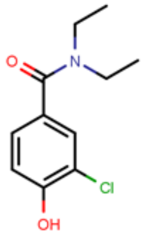

ligand 15

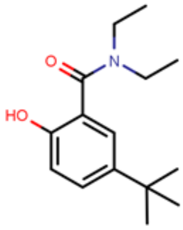

ligand 20

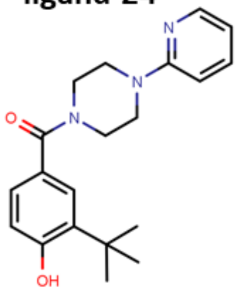

ligand 25

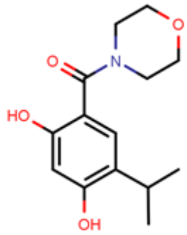

ligand 30

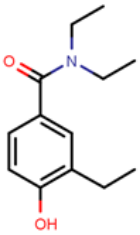

ligand 16

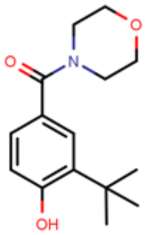

ligand 21

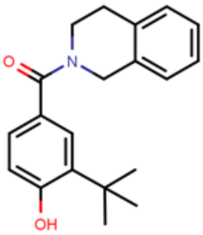

ligand 26

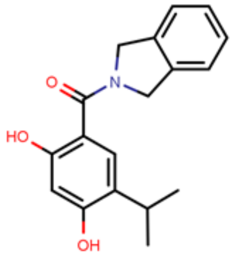

ligand 31

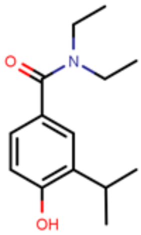

ligand 17

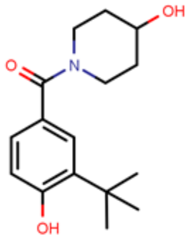

ligand 22

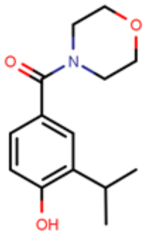

ligand 27

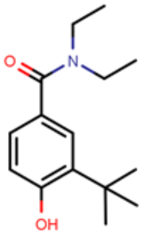

ligand 18

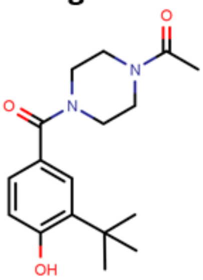

ligand 23

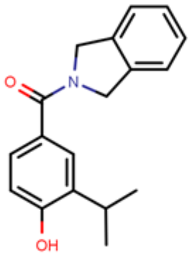

ligand 28

***Figure S2**. Structures of the 18 HSP90 ligands.*

Table S4. *Experimental and calculated binding free energies for the HSP90 ligands. ΔG^{ABFE} and ΔG^{MMGBSA} refers to the calculated ABFE and N$_{wat}$-MM/GBSA values respectively. All energies in kcal/mol.*

**Ligand ID**	**ΔG^{exp}**	**ΔG^{ABFE}**	**ΔG$^{MM/GBSA}$**
3	-4.23	-5.12 ± 0.55	-44.37 ± 0.47
15	-4.02	-5.98 ± 0.64	-41.82 ± 0.67
16	-5.93	-7.41 ± 0.76	-45.04 ± 0.89
17	-7.03	-9.93 ± 0.87	-48.70 ± 1.10
18	-6.91	-7.96 ± 0.63	-48.50 ± 0.81
19	-5.86	-9.21 ± 1.05	-49.53 ± 0.52
20	-5.28	-8.04 ± 1.93	-50.54 ± 0.80
21	-8.13	-12.68 ± 0.94	-52.80 ± 0.62
22	-7.69	-12.15 ± 0.70	-55.42 ± 0.74
23	-7.64	-12.53 ± 0.30	-55.91 ± 0.86
24	-9.01	-12.20 ± 0.71	-51.97 ± 1.02
25	-7.64	-13.00 ± 0.64	-58.76 ± 1.59
26	-8.73	-11.98 ± 1.01	-52.75 ± 1.32
27	-8.63	-12.06 ± 1.29	-50.97 ± 0.50
28	-9.78	-13.77 ± 1.05	-52.80 ± 0.61
29	-9.40	-13.38 ± 0.76	-51.49 ± 0.35
30	-10.86	-17.04 ± 0.77	-55.45 ± 0.46
31	-12.64	-18.28 ± 0.80	-58.18 ± 0.21

Table S5. *Experimental and calculated binding free energies (ΔG^{ABFE}) for HSP90 ligands using differing starting structures than those used in the main dataset results; 2XDL for ligands 21-27, 29-30, 2XHX with all three buried waters present for ligand 24, and the apo crystal 5J2V for ligand 31 . All energies in kcal/mol.*

**Ligand ID**	**ΔG^{exp}**	**ΔG^{ABFE}**	**Condition**
21	-8.13	-11.16 ± 1.07	2XDL
22	-7.69	-11.07 ± 0.82	2XDL
23	-7.64	-11.96 ± 1.17	2XDL
24	-9.01	-11.23 ± 0.88	2XDL
24	-9.01	-11.33 ± 0.52	2XHX - 3 waters
25	-7.64	-11.61 ± 0.78	2XDL
26	-8.73	-11.11 ± 1.22	2XDL
27	-8.63	-12.81 ± 0.54	2XDL
29	-9.40	-12.97 ± 0.91	2XDL
30	-10.86	-17.23 ± 0.79	2XDL
31	-12.64	-16.93 ± 0.92	5J2V

***Table S6.** Evaluation of the ligand elaboration steps of the HSP90 ligand set for the ABFE results. All energies in kcal/mol. *For the ligand 18 to 24 elaboration, we identify the free energy change to be in the wrong direction as ligand 24 should have the highest affinity when compared to ligands 25 and 26.*

Decision	Starting ligand ID(s)	Final ligand ID(s)	Elaboration outcome						
			ΔΔGexp	ΔΔGcalc	Sum(Error)	Expected outcome	ΔΔGcalc > Sum(Error)	Right direction?	Clear decision?
1	3	15	0.21	-0.77	1.19	No improvement	No	Yes	Yes
		16	-1.70	-2.20	1.31	Improved affinity	Yes	Yes	
		17	-2.80	-4.72	1.42		Yes	Yes	
		18	-2.68	-2.75	1.18		Yes	Yes	
2	17	19	1.17	0.72	1.92	Reduced affinity	No	Yes	No
	18	20	1.63	-0.08	2.56		No	No	
3	18	21	-1.22	-4.72	1.57	Improved affinty	Yes	Yes	Yes
		22	-0.78	-4.19	1.33		Yes	Yes	
		23	-0.73	-4.57	0.93		Yes	Yes	
4	18	24	-2.10	-5.29	1.34	Improved affinity	Yes	No*	No
		25	-0.73	-6.09	1.27		Yes	Yes	
		26	-1.82	-5.07	1.64		Yes	Yes	
5	21	27	-0.50	0.62	2.23	Improved affinity	No	No	No
	24	28	-0.77	-1.57	1.76		No	Yes	
	26	29	-0.67	-1.40	1.77		No	Yes	
6	27	30	-2.23	-4.98	2.06	Improved affinity	Yes	Yes	Yes
	28	31	-2.86	-4.51	1.85		Yes	Yes	

Table S7. *Evaluation of the ligand elaboration steps of the HSP90 ligand set for the N_{wat}-MM/GBSA results. All energies in kcal/mol.*

Decision	Starting ligand ID(s)	Final ligand ID(s)	Elaboration outcome						
			ΔΔGexp	ΔΔGcalc	Sum(Error)	Expected outcome	ΔΔGcalc > Sum(Error)	Right direction?	Clear decision?
1	3	15	0.21	1.55	1.14	No improvement	No	No	No
		16	-1.70	-0.67	1.36	Improved affinity	No	Yes	
		17	-2.80	-4.33	1.57		Yes	Yes	
		18	-2.68	-3.77	1.28		Yes	Yes	
2	17	19	1.17	-0.83	1.62	Reduced affinity	No	No	No
	18	20	1.63	-2.04	1.61		Yes	No	
3	18	21	-1.22	-4.30	1.43	Improved affinty	Yes	Yes	Yes
		22	-0.78	-6.92	1.55		Yes	Yes	
		23	-0.73	-7.41	1.67		Yes	Yes	
4	18	24	-2.10	-3.47	1.83	Improved affinity	Yes	No	No
		25	-0.73	-10.26	2.40		Yes	Yes	
		26	-1.82	-4.25	2.13		Yes	Yes	
5	21	27	-0.50	1.83	1.12	Improved affinity	Yes	No	No
	24	28	-0.77	-0.83	1.63		No	Yes	
	26	29	-0.67	1.26	1.67		No	No	
6	27	30	-2.23	-4.47	0.96	Improved affinity	Yes	Yes	Yes
	28	31	-2.86	-5.38	0.82		Yes	Yes	

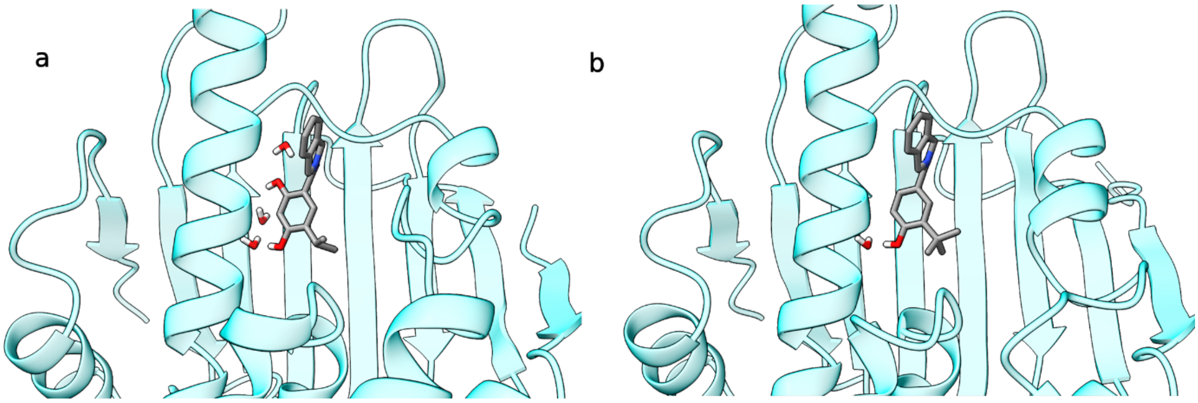

Figure S3. *Binding site waters of HSP90, a) ligand 31 bound to the 2XAB crystal conformation of HSP90 with all three buried waters present in the binding site, b) ligand 24 bound to the 2XHX crystal conformation of HSP90 with one binding site water.*

1.3 MCL-1

For this dataset we look at a set optimisations for two types of binders (termed class I and II) which occupy different parts of the MCL-1 binding site. The binders are then merged to form a higher affinity nanomolar compound (ligand 60). In this study, whilst we include the majority of the class I binders from Friberg *et al*.'s[3] work, we only include two class II compounds (ligands 16 and 17). This decision was made due to a) a lack of available structural data for ligands 18-20, and b) only ligands 16 and 17 being relevant to the formation of the merged ligand 60. We note that there are also several further optimisations of the merged ligands, however due to their non-fragment size we felt that these were out of scope of this study and therefore only one additional ligand from this optimisation set (ligand 65). All initial ligand poses were generated from the same protein-ligand complex crystal (PDB ID 4HW3).

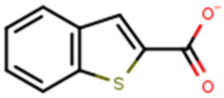

ligand 1

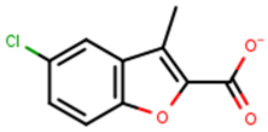

ligand 7

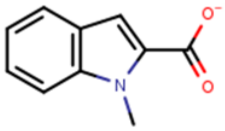

ligand 13

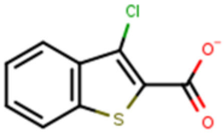

ligand 2

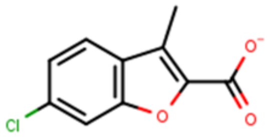

ligand 8

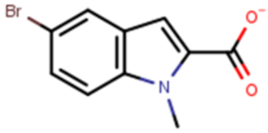

ligand 14

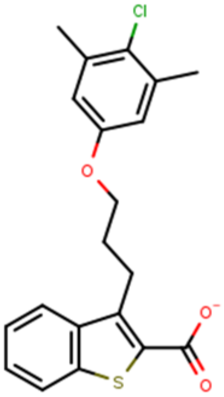

ligand 60

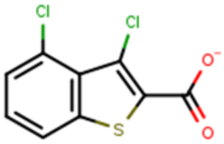

ligand 3

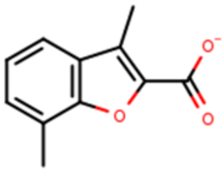

ligand 9

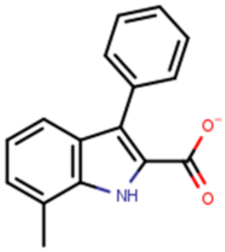

ligand 15

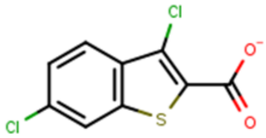

ligand 4

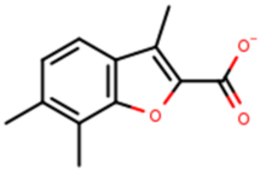

ligand 10

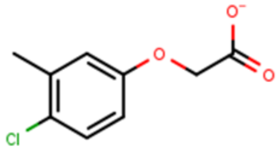

ligand 16

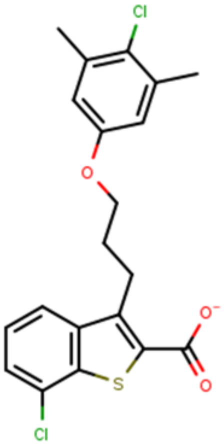

ligand 65

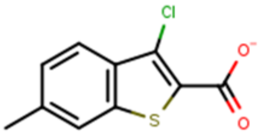

ligand 5

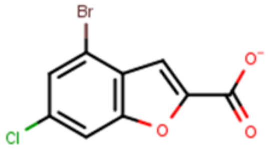

ligand 11

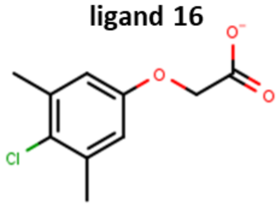

ligand 17

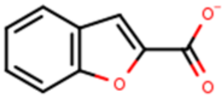

ligand 6

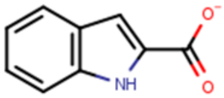

ligand 12

Figure S4*. Structures of the 19 MCL-1 ligands.*

Table S8. *Experimental and calculated binding free energies for the MCL-1 ligands. ΔG^{ABFE} and ΔG^{MMGBSA} refers to the calculated ABFE and N_{wat}-MM/GBSA values respectively. ΔG^{FEP+} represent FEP+ generated relative binding free energies obtained from Steinbrecher et al.'s fragment benchmark study[6]. All energies in kcal/mol.*

Ligand ID	ΔG^{exp}	ΔG^{ABFE}	$\Delta G^{MM/GBSA}$	ΔG^{FEP+}
1	-4.09	-5.61 ± 1.64	-37.03 ± 9.97	-4.49
2	-5.3	-8.23 ± 0.28	-43.16 ± 1.97	-6.08
3	-6.35	-10.53 ± 0.91	-47.58 ± 1.03	-7.42
4	-5.77	-9.07 ± 0.60	-42.65 ± 3.46	-6.27
5	-6	-7.75 ± 0.81	-44.66 ± 0.86	-5.71
6	-4.09	-5.19 ± 0.43	-35.70 ± 2.22	-3.25
7	-5.84	-7.36 ± 0.62	-40.29 ± 2.02	-5.03
8	-6.33	-8.26 ± 0.47	-41.40 ± 1.62	-6.37
9	-5.44	-8.02 ± 0.58	-42.59 ± 1.84	-6.52
10	-5.01	-8.14 ± 0.82	-44.08 ± 0.78	-6.15
11	-5.52	-9.21 ± 0.79	-43.14 ± 0.92	-6.26
12	-4.09	-4.26 ± 0.67	-38.33 ± 0.89	-1.96
13	-5.18	-5.52 ± 0.78	-41.75 ± 0.88	-3.88
14	-5.58	-7.41 ± 0.43	-44.42 ± 0.44	-5.4
15	-5.27	-6.94 ± 0.68	-45.39 ± 2.58	-5.58
16	-5.44	-7.68 ± 1.53	-44.72 ± 5.60	N/A
17	-5.76	-5.58 ± 1.11	-51.10 ± 4.66	N/A
60	-8.86	-10.64 ± 1.60	-62.93 ± 5.80	N/A
65	-8.36	-11.39 ± 1.84	-66.31 ± 1.65	N/A

Table S9. *Evaluation of the ligand elaboration steps of the MCL-1 ligand set for the ABFE results. All energies in kcal/mol.*

Decision	Starting ligand ID(s)	Final ligand ID(s)	Elaboration outcome						
			ΔΔGexp	ΔΔGcalc	Sum(Error)	Expected outcome	ΔΔGcalc > Sum(Error)	Right direction?	Clear decision?
1	1	2	-1.21	-2.62	1.92	Improved affinity	Yes	Yes	No
		3	-2.26	-4.92	2.55		Yes	Yes	
		4	-1.68	-3.46	2.24		Yes	Yes	
		5	-1.91	-2.14	2.46		No	Yes	
2	6	7	-1.75	-2.17	1.05	Improved affinity	Yes	Yes	Yes
		8	-2.24	-3.07	0.90		Yes	Yes	
		9	-1.35	-2.83	1.01		Yes	Yes	
		10	-0.92	-2.95	1.25		Yes	Yes	
		11	-1.43	-4.02	1.22		Yes	Yes	
3	12	13	-1.09	-1.26	1.45	Improved affinity	No	Yes	No
		14	-1.49	-3.15	1.10		Yes	Yes	
		15	-1.18	-2.68	1.35		Yes	Yes	
4	2	60	-3.56	-2.41	1.88	Improved affinity	Yes	Yes	Yes
	17		-3.10	-5.06	2.71		Yes	Yes	

Table S10. *Evaluation of the ligand elaboration steps of the MCL-1 ligand set for the N_{wat}-MM/GBSA results. All energies in kcal/mol.*

Decision	Starting ligand ID(s)	Final ligand ID(s)	Elaboration outcome						
			ΔΔGexp	ΔΔGcalc	Sum(Error)	Expected outcome	ΔΔGcalc > Sum(Error)	Right direction?	Clear decision?
1	1	2	-1.21	-6.13	11.94	Improved affinity	No	Yes	No
		3	-2.26	-10.13	11.00		No	Yes	
		4	-1.68	-5.62	13.43		No	Yes	
		5	-1.91	-7.63	10.83		No	Yes	
2	6	7	-1.75	-4.59	4.24	Improved affinity	Yes	Yes	Yes
		8	-2.24	-5.70	3.84		Yes	Yes	
		9	-1.35	-6.89	4.06		Yes	Yes	
		10	-0.92	-8.38	3.00		Yes	Yes	
		11	-1.43	-7.44	3.14		Yes	Yes	
3	12	13	-1.09	-3.42	1.77	Improved affinity	Yes	Yes	Yes
		14	-1.49	-6.09	1.33		Yes	Yes	
		15	-1.18	-7.06	3.47		Yes	Yes	
4	2	60	-3.56	-19.77	7.77	Improved affinity	Yes	Yes	Yes
	17		-3.10	-11.83	10.46		Yes	Yes	

Table S11. *Evaluation of the ligand elaboration steps of the MCL-1 ligand set based on the FEP+ RBFE results as obtained from Steinbrecher et al.'s fragment benchmark study[6]. Analysis of errors is not provided as specific error bars were not provided in the original study. All energies in kcal/mol.*

Decision	Starting ligand ID(s)	Final ligand ID(s)	Elaboration outcome				
			ΔΔGexp	ΔΔGcalc	Expected outcome	Right direction?	Clear decision?
1	1	2	-1.21	-1.59	Improved affinity	Yes	Yes
		3	-2.26	-2.93		Yes	
		4	-1.68	-1.78		Yes	
		5	-1.91	-1.22		Yes	
2	6	7	-1.75	-1.78	Improved affinity	Yes	Yes
		8	-2.24	-3.12		Yes	
		9	-1.35	-3.27		Yes	
		10	-0.92	-2.90		Yes	
		11	-1.43	-3.01		Yes	
3	12	13	-1.09	-1.92	Improved affinity	Yes	Yes
		14	-1.49	-3.44		Yes	
		15	-1.18	-3.62		Yes	

1.4 Cyclophilin D

For Cyclophilin D we look at a set of fragment merging decisions by Grädler *et al.*[4] These merges aim to combine fragments occupying distinct binding sites in the Cyclophilin D structure, optimising ligands from millimolar to nanomolar affinities. Whilst the original study looks at a total of 40 ligands, we have chosen to instead select a smaller sub-selection of ten ligands. These were specifically chosen as they had more readily available structural data. Several X-ray crystals were chosen as starting points for the different ligands; 6R8O (ligands 2 and 14), 6R9S (ligands 3, 27, and 39), 6R9U (ligand 4), 6RA1 (ligands 7 and 39), 6R9X (ligands 8 and 27), 6R8L (ligand 16). Ligands 27 and 39 were generated from the overlap of two crystal structures.

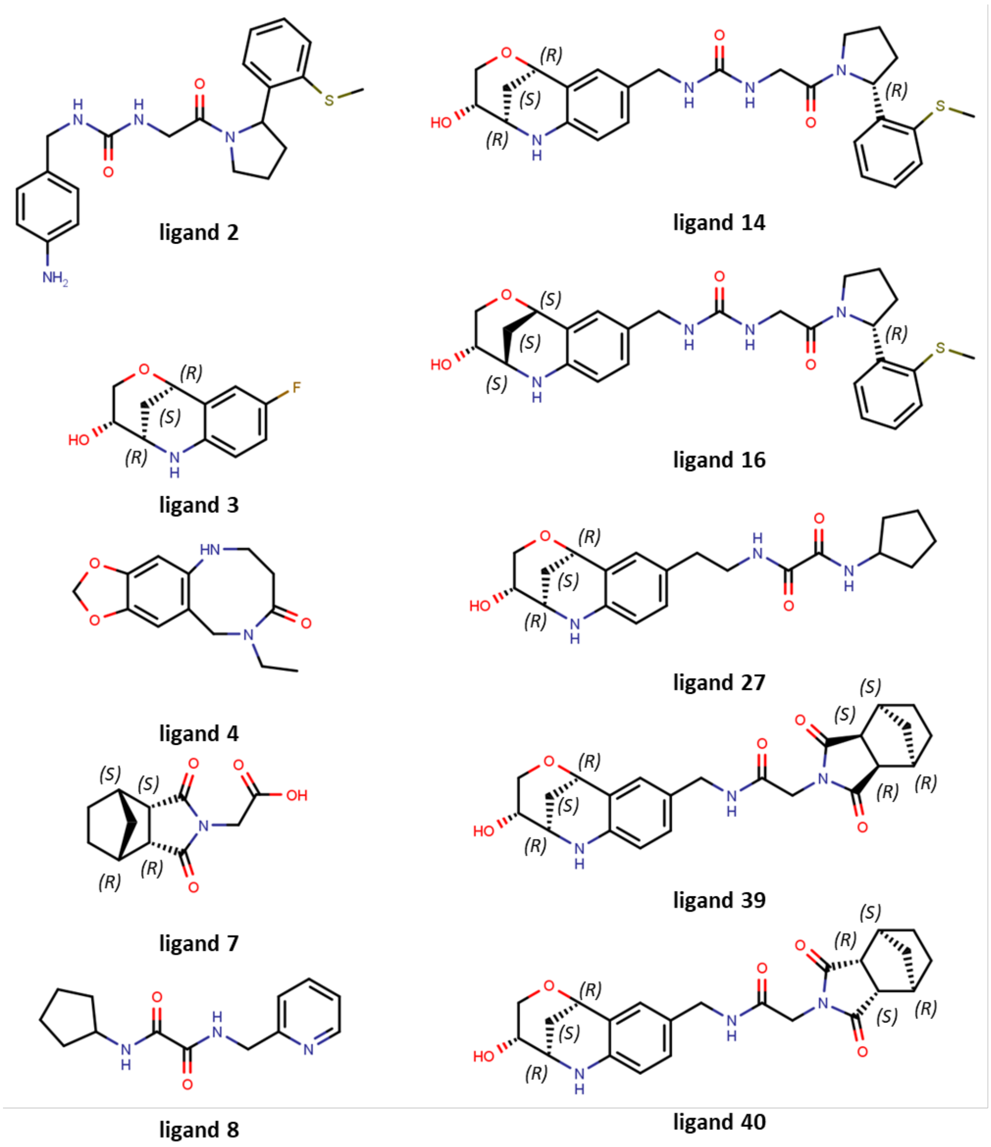

***Figure S5**. Structures of the 10 Cyclophilin D ligands. Structures adapted from Grädler et al.*[4]

Table S12. *Experimental and calculated binding free energies for the Cyclophilin D ligands. ΔG^{ABFE} and ΔG^{MMGBSA} refers to the calculated ABFE and N_{wat}-MM/GBSA values respectively. All energies in kcal/mol. *An extra entry for ligand 27 is shown demonstrating the calculated ABFE obtained when starting from the apo crystal 3QYU instead of PDB 6R9S.*

**Ligand ID**	ΔG^{exp}	ΔG^{ABFE}	$\Delta G^{MM/GBSA}$
2	-9.06	-8.18 ± 0.61	-54.89 ± 0.21
3	-2.93	-4.71 ± 0.22	-33.51 ± 0.13
4	-2.9	-4.14 ± 0.81	-35.12 ± 1.67
7	-2.73	-4.85 ± 0.17	-58.05 ± 0.41
8	-4.04	-7.24 ± 0.59	-37.24 ± 0.82
14	-11.22	-12.92 ± 0.43	-69.51 ± 0.47
16	-8.42	-10.54 ± 0.48	-65.55 ± 2.72
27	-7.57	-10.21 ± 1.10	-61.70 ± 0.67
27 – 3QYU*	-7.57	- 10.50 ± 1.04	-
39	-8.43	-12.62 ± 0.47	-67.44 ± 0.23
40	-8.08	-11.78 ± 0.52	-64.79 ± 0.38

Table S13. *Evaluation of the ligand elaboration steps of the Cyclophilin D ligand set for the ABFE results. All energies in kcal/mol*.

Decision	Starting ligand ID(s)	Final ligand ID(s)	Elaboration outcome						
			ΔΔGexp	ΔΔGcalc	Sum(Error)	Expected outcome	ΔΔGcalc > Sum(Error)	Right direction?	Clear decision?
1	2	14	-2.16	-4.74	1.04	Improved affinity	Yes	Yes	Yes
	3		-8.29	-8.21	0.65		Yes	Yes	
2	2	16	0.64	-2.36	1.09	Improved affinity	Yes	No	No
	3		-5.49	-5.83	0.70		Yes	Yes	
3	3	27	-4.64	-5.50	1.32	Improved affinity	Yes	Yes	Yes
	8		-3.53	-2.97	1.69		Yes	Yes	
4	3	39	-5.50	-7.91	0.69	Improved affinity	Yes	Yes	Yes
	7		-5.70	-7.77	0.64		Yes	Yes	
5	3	40	-5.15	-7.07	0.74	Improved affinity	Yes	Yes	Yes
	7		-5.35	-6.93	0.69		Yes	Yes	

Table S14. *Evaluation of the ligand elaboration steps of the Cyclophilin D ligand set for the Nwat-MMGBSA results. All energies in kcal/mol.*

Decision	Starting ligand ID(s)	Final ligand ID(s)	Elaboration outcome						
			ΔΔGexp	ΔΔGcalc	Sum(Error)	Expected outcome	ΔΔGcalc > Sum(Error)	Right direction?	Clear decision?
1	2	14	-2.16	-14.62	0.68	Improved affinity	Yes	Yes	Yes
	3		-8.29	-36.00	0.68		Yes	Yes	
2	2	16	0.64	-10.66	2.93	Improved affinity	Yes	No	No
	3		-5.49	-32.04	2.84		Yes	Yes	
3	3	27	-4.64	-28.19	0.79	Improved affinity	Yes	Yes	Yes
	8		-3.53	-24.46	1.49		Yes	Yes	
4	3	39	-5.50	-33.93	0.35	Improved affinity	Yes	Yes	Yes
	7		-5.70	-9.40	0.64		Yes	Yes	
5	3	40	-5.15	-31.28	0.50	Improved affinity	Yes	Yes	Yes
	7		-5.35	-6.75	0.79		Yes	Yes	

Supplementary Note S2. PWWP1 N-termini

The PWWP1 domain construct used in the Böttcher *et al.* [1] study comprises of amino acids 247 to 398 of NSD3. This construct contains a disordered N-termini region starting approximately at PHE 392. Initial simulations of this construct on selected ligands (8, 12, 16, and 17) demonstrated this termini region to be quite flexible, adopting a range of various conformations (**Figure S6**). In particular, we find that in some cases the N-termini can adopt conformations which interact directly with the binding site. This is generally involves interactions with ASP 396, particularly in the case of charge ligands (i.e. ligands **12** and **17**), where we see a higher propensity for interactions between the N-termini and ligand. Sufficiently sampling these N-termini motions would require much longer timescales than are possible with the 20 ns sampling windows used in our ABFE protocol. Combined with concerns regarding the propensity for certain AMBER force fields to overly prefer compact structures [7], we instead used a model with a shorter N-termini which is truncated at ILE 393 and thus avoids interactions with the binding site.

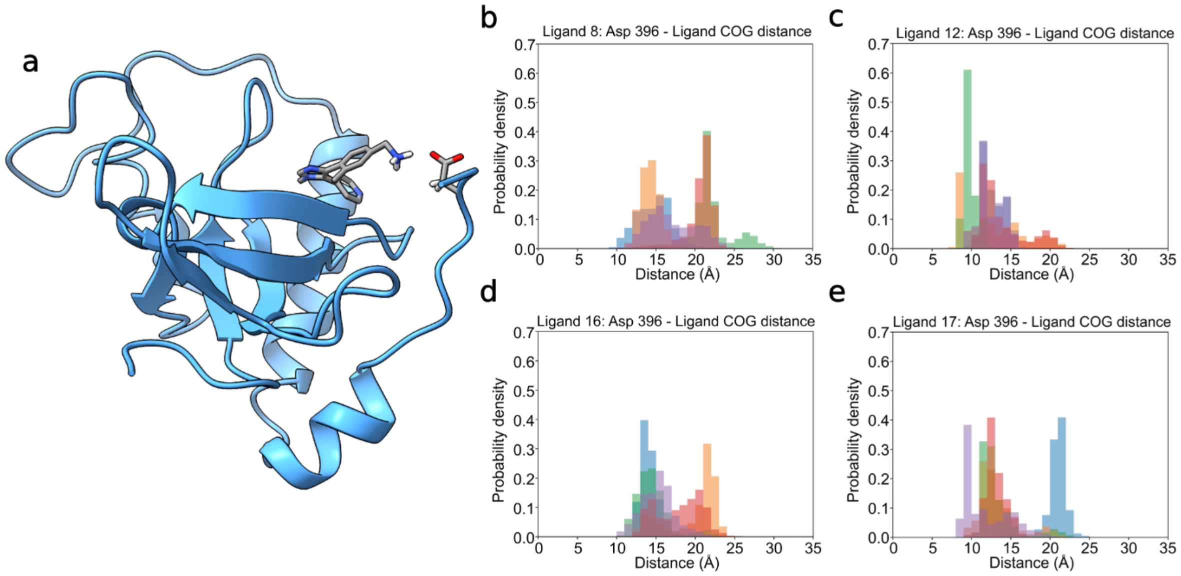

Figure S6. *Interactions between PWWP1 construct N-termini ASP 396 and the ligand, a) representative frame demonstrating the formation of a close contact between the charged ligand 12 and ASP 396, b-e) plots of the centre of geometry distances between ASP 396 and ligands 8, 12, 16, and 17 respectively. Distances in Angstroms.*

Supplementary Note S3. Evaluation of HMR in GROMACS ABFE calculations

In order to provide a short evaluation of the use HMR in GROMACS ABFE calculations, we calculated the Cyclophilin D dataset using both standard masses (2 fs timestep), and HMR (4 fs timestep) (**Figure S7**). The Cyclophilin D dataset was chosen due to having both one of the larger ligand structural diversities amongst our datasets and also being rather small and much faster to simulate (approximately 33 000 atoms once solvated). As we can see from **Figure S7**, for the most part the free energies agree well with deviations in the estimates remaining within error. In only two cases do we see a greater than 1 kcal/mol difference in the ΔΔG, ligands 2 and 39 (ΔΔG of 1.06 and 1.19 kcal/mol respectively). In the case of ligand 39, we also have the only pair which shows a greater than error difference between the two methods. This difference between the estimates is likely to be caused by limitations in sampling, indeed expanding the number of replicas to 20 for both ligands 2 and 39 reduces the ΔΔG to 0.51 and 1.05 kcal/mol respectively. In the case of ligand 39, the difference in the estimates remains rather large but now lies within the range of error of each estimate.

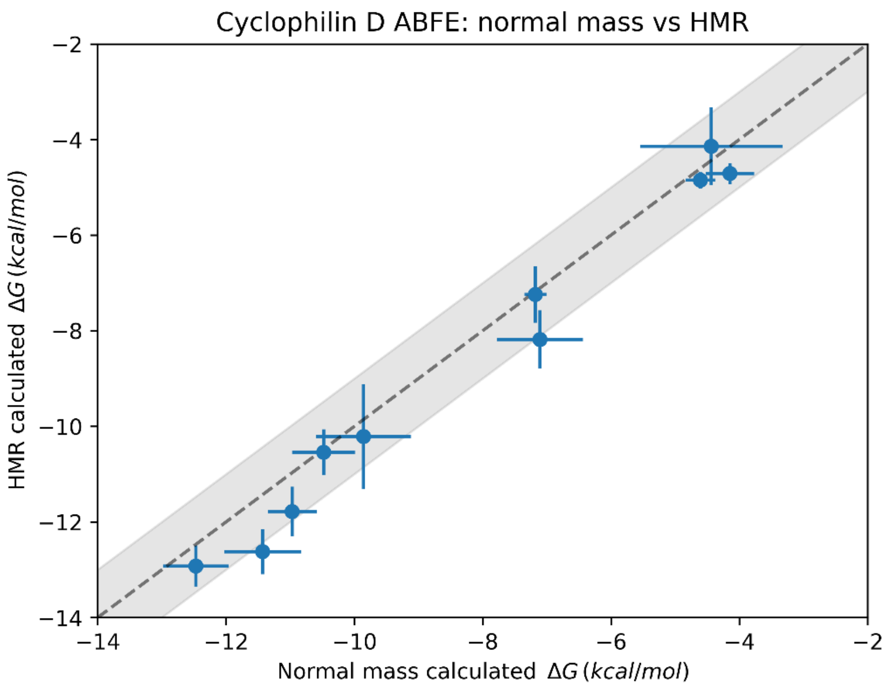

Figure S7. *Comparison of normal mass and HMR ABFE calculations for the Cyclophilin D dataset. Free energies in kcal/mol. Free energy estimates are the means of the estimates across replicas, with error bars as their standard deviation.*

Table S15. *Experimental and calculated binding free energies for the Cyclophilin D ligands. $\Delta G^{ABFE\text{-}HMR}$ and $\Delta G^{ABFE\text{-}NORM}$ refers to the calculated ABFE values using Hydrogen Mass Repartitioning and standard masses respectively. Additional entries are provided for ligands 2 and 39 for which extra replicas were calculated to evaluate the convergence between the HMR and NORM results. All energies in kcal/mol.*

Ligand ID	ΔG^{exp}	$\Delta G^{ABFE\text{-}HMR}$	$\Delta G^{ABFE\text{-}NORM}$
2	-9.06	-8.18 ± 0.61	-7.11 ± 0.67
2 (20 repeats)	-9.06	-7.69 ± 0.75	-7.18 ± 0.66
3	-2.93	-4.71 ± 0.22	-4.15 ± 0.38
4	-2.9	-4.14 ± 0.81	-4.44 ± 1.11
7	-2.73	-4.85 ± 0.17	-4.61 ± 0.27
8	-4.04	-7.24 ± 0.59	-7.18 ± 0.17
14	-11.22	-12.92 ± 0.43	-12.47 ± 0.51
16	-8.42	-10.54 ± 0.48	-10.48 ± 0.49
27	-7.57	-10.21 ± 1.10	-9.86 ± 0.74
39	-8.43	-12.62 ± 0.47	-11.43 ± 0.60
39 (20 repeats)	-8.43	-12.49 ± 0.58	-11.43 ± 0.55
40	-8.08	-11.78 ± 0.52	-10.97 ± 0.38

Supplementary Note S4. Correlation analyses of signed errors between ABFE, MMGBSA, and FEP+

In order to evaluate whether similar trends are seen in the deviations from experiment between methods, an analysis of the correlation of signed errors is shown here.

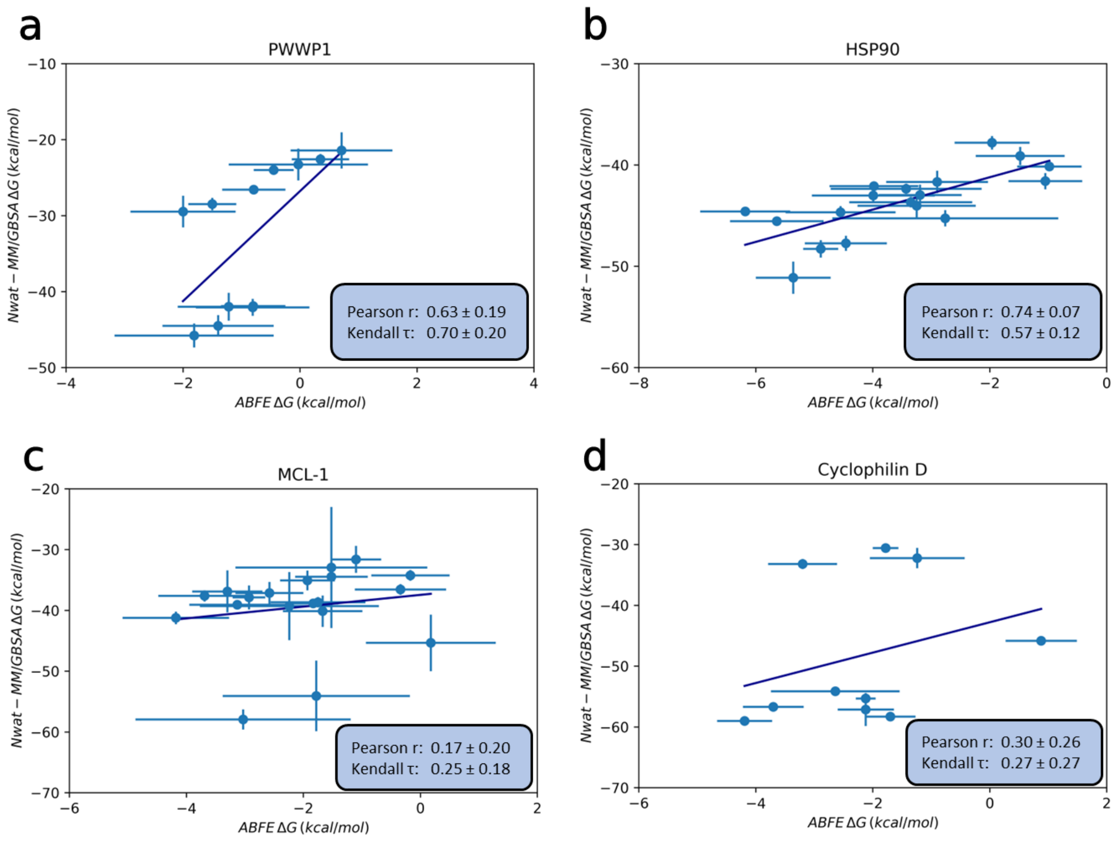

Figure S8. *Analyses of the correlation of signed errors between ABFE and N_{wat}-MM/GBSA for the a) PWWP1, b) HSP90, c) MCL-1, and d) Cyclophilin D datasets. Free energy estimates are the means of the estimates across replicas, with error bars as their standard deviation. Correlation metrics calculated from the mean estimate values, with error bars derived from bootstrap resampling. All free energy results, including RMSE values, have units of kcal/mol.*

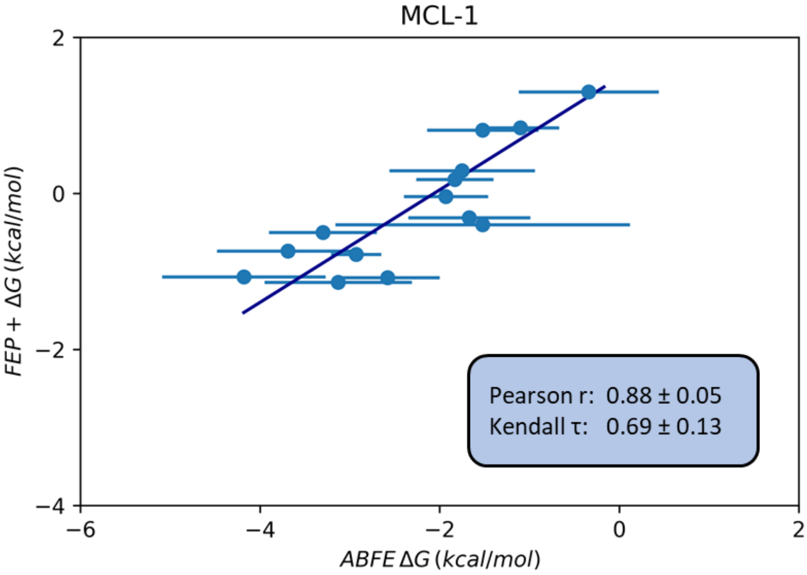

Figure S9. *Analysis of the correlation of signed errors between ABFE and FEP+ for the MCL-1 dataset.* Free energy estimates are the means of the estimates across replicas, with error bars as their standard deviation. Correlation metrics calculated from the mean estimate values, with error bars derived from bootstrap resampling. All free energy results have units of kcal/mol.

Supplementary Note S5. Outlier analyses

In order to identify potential outliers in our calculated results, Random Sample Consensus (RANSAC)[8] analyses were carried out using the RANSAC Regressor algorithm implemented in the scikit-learn library. The minimum number of samples to be evaluated was set at half the number of ligands in the dataset and a maximum number of 1 000 000 trials were allow for random sample selection. We note that error bars were not included as part of this outlier analysis.

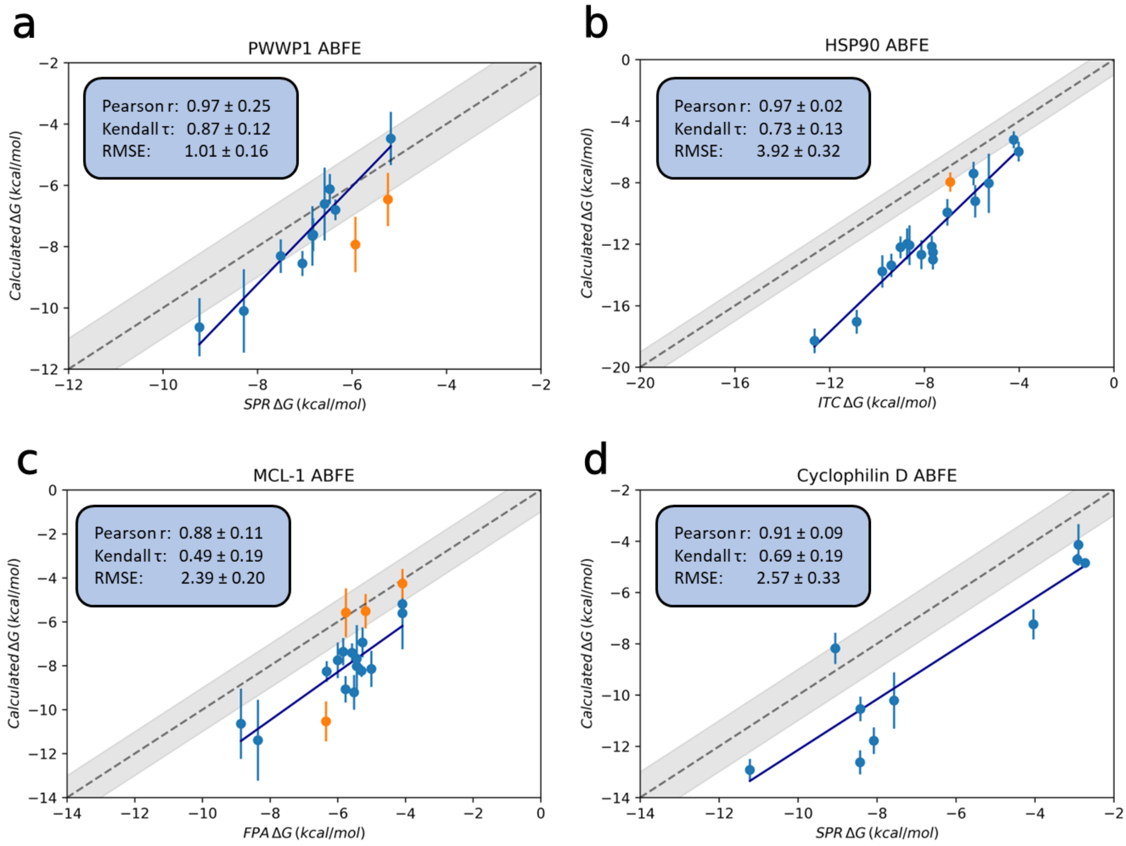

Figure S10. *RANSAC outlier analysis of the ABFE results for the a) PWWP1 [outliers: ligands 15 and BI-9466], b) HSP90 [outliers: ligand 18], c) MCL-1 [outliers: ligands 3, 12, 13, and 17], and d) Cyclophilin D [no outliers detected] datasets. Pearson r, Kendall τ and RMSE are reported excluding outliers. Free energy estimates are the means of the estimates across replicas, with error bars as their standard deviation. Correlation metrics calculated from the mean estimate values, with error bars derived from bootstrap resampling. All free energy results, including RMSE values, have units of kcal/mol.*

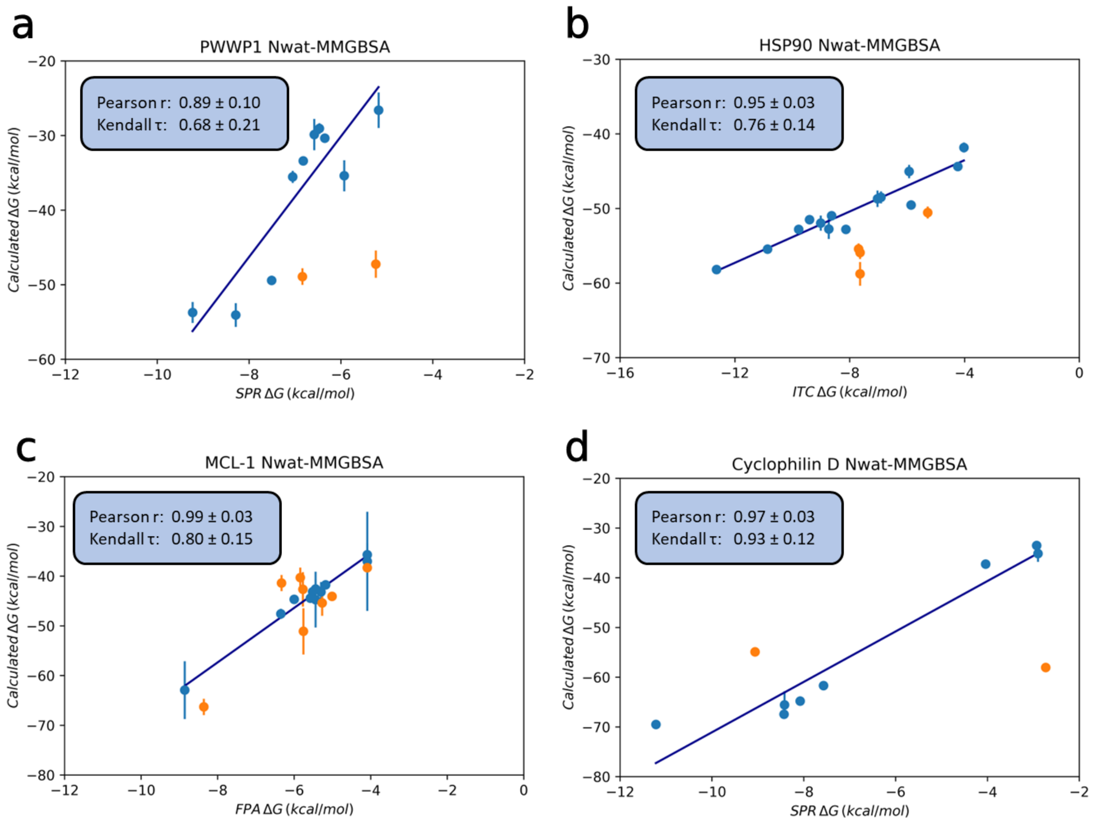

Figure S11. *RANSAC outlier analysis of the N_{wat}-MM/GBSA results for the a) PWWP1 [outliers: ligands 13 and BI-9466], b) HSP90 [outliers: ligands 20, 22-24], c) MCL-1 [outliers: ligands 4, 7, 8, 10, 12, 15, 17, 65], and d) Cyclophilin D [outliers: ligands 2, 7] datasets. Pearson r, and Kendall τ are reported excluding outliers. Free energy estimates are the means of the estimates across replicas, with error bars as their standard deviation. Correlation metrics calculated from the mean estimate values, with error bars derived from bootstrap resampling. All free energy results, have units of kcal/mol.*

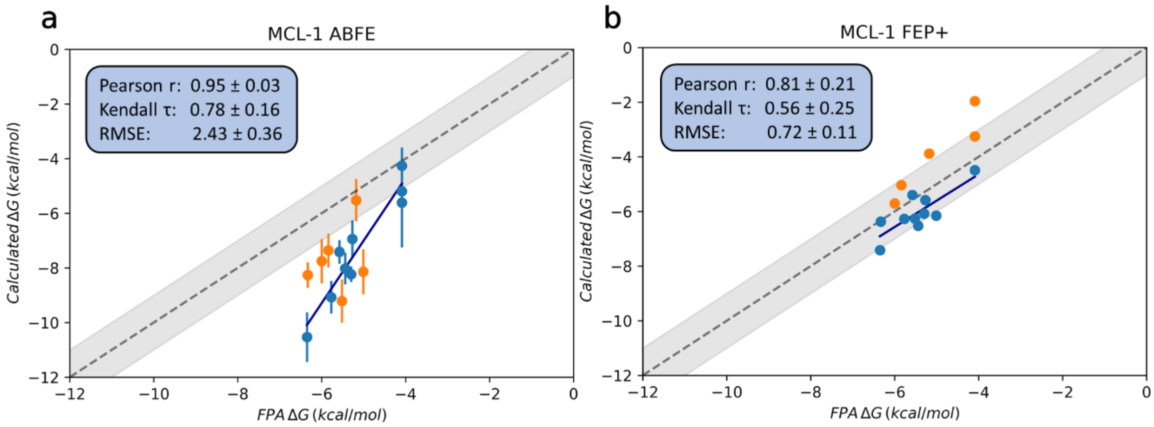

Figure S12. *RANSAC outlier analysis of the ABFE [outliers: 3, 5, 7, 8, 10, 13] and FEP+ [outliers: 1, 2, 7, 9, 10, 12, 13] results for the MCL-1 datasets. Pearson r, Kendall τ and RMSE are reported excluding outliers. Free energy estimates are the means of the estimates across replicas, with error bars as their standard deviation. Correlation metrics calculated from the mean estimate values, with error bars derived from bootstrap resampling. All free energy results, including RMSE values, have units of kcal/mol.*

Supplementary Note S6. Redocking of PWWP1 ligands

Due to their weak binding nature, fragments often tend to occupy multiple binding poses. In this work our datasets had for the most part known binding poses, therefore the issue of identifying the correct pose was avoided. However, in practice this may not always be feasible. Therefore, one may envision potentially using ABFE calculations in order to discriminate between different fragment poses by identifying native poses as the most favourable binders. Indeed, such an approach has been shown to work for larger ligands[9]. Here we carry out a preliminary investigation on the feasibility of using ABFEs as a means to discriminate native poses for fragments by redocking two ligands from the PWWP1 dataset.

Ligands 8 and 16 (**SI Fig. S1**) were redocked into the PWWP1 binding site using smina [10,11] (9th November, 2017 release) using a 20 Å search grid centred around the centre of geometry of the native ligand pose. From these docking poses, 3 diverse poses were obtained from the top 10 ligands and a single ABFE calculation was carried out for each redocked pose. Results for the ligand in solvent portion of the cycle were taken from the first replica of the previously computed native pose ligand ABFE.

Comparing these ABFE results with those of the native poses (**SI Table S1**) we find that whilst the native pose is the one with the strongest predicted binding energy for ligand 16, this is the not the case of ligand 8. Indeed, the third redocked pose of ligand 8 is predicted to have a stronger affinity by more than 3 kcal/mol compared to the native pose. This result is not too surprising in that ligand 16 is larger than ligand 8.

Due to the very limited number of data points calculated here, and the lack of repeats, it is not possible to make strong conclusions on how well this methodology could identify native fragment poses. However, the inability to readily identify the correct pose for ligand 8 does indicate that more work, and possibly more sophisticated sampling methods, may be required to accurately use ABFEs for this purpose and confirms that this is particularly challenging.

Table S16. *Heavy atom RMSDs from native pose for both the initial redocked pose and the post molecular dynamics equilibration pose and calculated ABFE results for the redocked poses for ligands 8 and 16 of the PWWP1 dataset. RMSD in Å and energies in kcal/mol.*

Ligand ID	Initial heavy atom RMSD	Post equilibration heavy atom RMSD	ΔG^{ABFE}
8	5.92	4.97	-3.30
8	4.86	1.63	-4.56
8	5.52	4.10	-8.16
16	4.70	4.02	-5.00
16	5.21	5.53	-6.67
16	5.45	4.55	-4.26

Supplementary References

1 Böttcher, J. *et al.* Fragment-based discovery of a chemical probe for the PWWP1 domain of NSD3. *Nature Chem. Biol.* **15**, 822-829, doi:10.1038/s41589-019-0310-x (2019).

2 Murray, C. W. *et al.* Fragment-based drug discovery applied to HSP90. Discovery of two lead series with high ligand efficiency. *J. Med. Chem.* **53**, 5942-5955, doi:10.1021/jm100059d (2010).

3 Friberg, A. *et al.* Discovery of potent myeloid cell leukemia 1 (Mcl-1) inhibitors using fragment-based methods and structure-based design. *J. Med. Chem.* **56**, 15-30, doi:10.1021/jm301448p (2013).

4 Grädler, U. *et al.* Discovery of novel Cyclophilin D inhibitors starting from three dimensional fragments with millimolar potencies. *Bioorg. Med. Chem. Letts* **29**, 126717, doi:https://doi.org/10.1016/j.bmcl.2019.126717 (2019).

5 The PyMOL molecular graphics system. (2004).

6 Steinbrecher, T. B. *et al.* Accurate binding free energy predictions in fragment optimization. *J. Chem. Inf. Model.* **55**, 2411-2420, doi:10.1021/acs.jcim.5b00538 (2015).

7 Tian, C. *et al.* ff19SB: Amino-acid-specific protein backbone parameters trained against quantum mechanics energy surfaces in solution. *J. Chem. Theory Comp.* **16**, 528-552, doi:10.1021/acs.jctc.9b00591 (2020).

8 Fischler, M. A. & Bolles, R. C. Random sample consensus: A paradigm for model fitting with applications to image analysis and automated cartography. *Comm. ACM* **24**, 381-395 (1981).

9 Aldeghi, M., Heifetz, A., Bodkin, M. J., Knapp, S. & Biggin, P. C. Accurate calculation of the absolute free energy of binding for drug molecules. *Chem. Sci.* **7**, 207-218, doi:10.1039/C5SC02678D (2016).

10 Trott, O. & Olson, A. J. AutoDock Vina: improving the speed and accuracy of docking with a new scoring function, efficient optimization, and multithreading. *J. Comput. Chem.* **31**, 455-461, doi:10.1002/jcc.21334 (2010).

11 Koes, D. R., Baumgartner, M. P. & Camacho, C. J. Lessons learned in empirical scoring with smina from the CSAR 2011 benchmarking exercise. *J. Chem. Inf. Model.* **53**, 1893-1904, doi:10.1021/ci300604z (2013).
