# Supplementary material for: Evaluating the use of absolute binding free energy in the fragment optimisation process
Source: Commun Chem. 2022 Sep 5;5:105. doi: 10.1038/s42004-022-00721-4 (PMC9814858; doi:10.1038/s42004-022-00721-4)
Supplement: Supplementary file 1 — Biggin_PR File [file 42004_2022_721_MOESM1_ESM.pdf]

nature portfolio

Peer Review File

Evaluating the use of Absolute Binding Free Energy in the fragment optimization process

Open Access This file is licensed under a Creative Commons Attribution 4.0 International License, which permits use, sharing, adaptation, distribution and reproduction in any medium or format, as long as you give appropriate credit to the original author(s) and the source, provide a link to the Creative Commons license, and indicate if changes were made. In the cases where the authors are anonymous, such as is the case for the reports of anonymous peer reviewers, author attribution should be to 'Anonymous Referee' followed by a clear attribution to the source work. The images or other third party material in this file are included in the article's Creative Commons license, unless indicated otherwise in a credit line to the material. If material is not included in the article's Creative Commons license and your intended use is not permitted by statutory regulation or exceeds the permitted use, you will need to obtain permission directly from the copyright holder. To view a copy of this license, visit http://creativecommons.org/licenses/by/4.0/.

Reviewers' comments:

Reviewer #1 (Remarks to the Author):

The authors present a validation of the use of absolute binding free energy calculations (ABFEP) for fragment optimization. The paper covers 4 systems, some of which have been extensively studied in the computational chemistry field in the past.
A workflow based on the Amber and Gromacs packages is described that prepares systems and simulates them using a well established ABFEP protocol, including defining proper restraints for convergence reasons. Calculations then show that compound binding affinities can be predicted with good correlation, but moderately high errors, compared to experiment. This shows that ABFEP can play a role in fragment optimization campaign, but some uncertainties about the practical use of the method remain.

Alltogether, the paper is well written and publishes convincing data on a relevant area of the field. The results are overall not very surprising, as the authors themselves cite, ABFEP calculations on fragments are not a novel idea, generally expected to work and that is what their data shows as well. Nevertheless, presenting data for multiple systems and a detailed description of their workflow should make this paper of interest for researchers engaged in fragment based design or free energy calculations. I'd therefore recommend publication of the manuscript.

One thing that I felt was missing from and could be added to the discussion is the influence of receptor holo-conformations on the results. The authors note the regular overprediction of ABFEP dG values compared to experiment clearly visible in Fig 3b-e. Since ABFEP calculations decouple ligands from a binding pocket, but do not give the binding pocket time to fully relax to its apo-state, the free energies would need to be corrected by this receptor strain contribution. In practice, this is difficult to do and not relevant for actual design, since even with ABFEP only the relative ranking of molecules matters. Therefore I believe it would be fair to adjust the computed results (e.g. with a constant offset that minimizes computed dG error compared to experiment, like it is done for RBFEP) for that and both present the relevant statistics for these dG* results as well as discuss the amount of offset needed as an indication of receptor strain.

The authors note a limitation of their results, namely that their accurate scoring depends on knowing the right bound pose of the fragments. This is not realistic in a practical applications setting, but this concern could be eased by running a few ABFEP calculations with 'wrong' poses. Surely, many fragments could be flipped in the pocket or redocked into other parts of the receptor pocket. If ABFEP were able to pick out the 'correct' bound pose among these, a major limitation of the presented method would be removed.

There seems to be a mangled sentence on page 11 "...decision occurs when in that set..."

I think giving RMSE values for MMGBSA is not a fair assessment of the method performance, MMGBSA is not usually expected to give quantitative scores in this fashion and such errors are always huge. To compute a meaningful RMSE, one would at least have to apply the same constant offset shifting to match experimental averages as suggested above or done in RBFEP.

Reviewer #2 (Remarks to the Author):

In Alibay et al, authors describe the application and performance of Absolute Binding Free Energy (ABFE) calculations in guiding fragment optimization decisions. The authors retrospectively calculated the binding free energies of 59 ligands to four previously published Fragment-Based Drug Design (FBDD) campaigns, each with a different protein target. They investigated whether ABFE calculations can predict experimental in vitro affinity results and whether they can clearly support synthesis

decisions in the fragment optimization process. Finally, they compared their results against cheaper endpoint methods, namely Nwat-MM/GBSA. Results suggest that ABFE offer improved free energy estimations compared to Nwat-MM/GBSA. The work carried out is important and I believe that the application of ABFE calculations will be useful for the field. In addition, the manuscript is clearly written, providing reproducible calculations, and thoroughly report and analyse the results. Therefore, the paper should be published with minor revisions. I have only minor comments and suggestions to improve the clarity of the presentation.

1) Main text, Page 9: It is mentioned that five independent replicas are simulated for each ABFE simulation using different orientational restraints. The selection of the orientational restraints is performed using MDRestraintsGenerator tool in the final 20 ns NPT equilibration simulation. The frame closest to the mean bond, angle and dihedral values of the chosen restraint over the 20 ns simulation is used as the starting point of the ABFE simulations. Based on the selection of the restraints it would be helpful if authors state whether they also use different starting points for each replica. If not, could the authors explain how they pick the rest of the orientational restraints that they are using in the ABFE calculations?

2) Main text, Page 12 and Page 18: In Figures 3 and 6, the experimental technique for each FBDD set is written in the x-axis except for the MCL-1 dataset. Could the authors add the experimental technique for this dataset as well?

3) Main text, Page 17: The authors discuss a systematic shift in free energy of the Cyclophilin D dataset by approximately 2.5 kcal/mol. Could the authors provide a possible explanation for this shift, as they have done in the other datasets?

4) Main text, Page 17: The authors claim that the 6 nM ligand 16, is estimated as within 0.3 kcal/mol of the 660 nM ligand 39. However, based on Table S10, we can observe that ligands 16 and 39 have similar experimental values (ΔG_{exp} = -8.42 kcal/mol for ligand 16 and ΔG_{exp} = -8.43 kcal/mol for ligand 39). In addition, the reported binding free energies based on ABFE calculations are not within 0.3 kcal/mol (ΔG_{ABFE} = -10.54 ± 0.48 kcal/mol for ligand 16 and ΔG_{ABFE} = -12.62 ± 0.47 kcal/mol for ligand 39). Could the authors kindly provide an explanation for this claim?

5) Main text, Page 19: "We also show that ABFE calculations ………………….. offer comparable results to relative binding free energy methods". It would be nice if the authors can also add in Supplementary Information, a table containing an evaluation of the ligand elaboration steps of the MCL-1 ligand set for the RBFE calculations performed by Steinbrecher et al. In this way, they will be also to compare the predictive ability of relative binding free energy methods compared to ABFE calculations.

6) Supporting Information, Page 5: In Table S2, ligand 14 is not present in the ligand elaboration steps of the PWWP1 ligand set. It would be helpful for the reader to point out why this ligand was included in the ABFE calculations since it is not present in the fragment optimization process.

7) Supporting Information, Page 9: In elaboration decision 4, 18-24 perturbation yields better improvement in affinity for both experimental and computational techniques. Could the authors explain why they have placed "No" in the Right direction section?

8) Supporting Information, Page 12: Authors should add a negative sign to the acid groups in Figure S3. This will demonstrate the charged nature of the ligands.

9) Supporting Information, Page 13: In Table S7, ligand 1 is predicted to have a positive binding free energy. I believe that this is probably a typo (based on Figure 3 in the main text), but can the authors provide an explanation for this behaviour if this is not the case?

10) Supporting Information, Page 20: The authors provide an evaluation of the use of hydrogen mass

repartitioning scheme (HMR) in GROMACS ABFE calculations, using both standard masses and HMR. It would be useful for the reader, if authors add a table with the predicted binding free energies for both schemes, using 5 and 20 replicas.

11) Typos:

- Main text, Page 8, Fig 2: Replace “ligand electrostatic” with electrostatics.

- Main text, Page 11: References for the 4 FBDD sets have been placed again as 89, 90, 91, 92.

- Supporting Information, Page 13: In Table S10, replace “HSP90” with “Cyclophilin D”.

Reviewer #3 (Remarks to the Author):

In “Evaluating the use of Absolute Binding Free Energy in the fragment optimization process,” the authors assess the ability of Absolute Binding Free Energy (ABFE) calculations to make fragment memory optimization decisions. They test ABFE performance for fragments across four different optimization campaigns and present potential issues with HSP90 and MCL-1. They further compare ABFE simulations to Nwat-MM/GBSA and RBFE FEP+. They found that while somewhat computationally demanding, ABFE outperforms Nwat-MM/GBSA and that ABFE can successfully be used to make fragment optimization decisions.

Fragment optimization is particularly appealing because understanding how individual fragments alter affinity allows for a coarse graining of the chemical space of potential active binders. But, as noted by the authors, fragment affinity is relatively low, creating both an experimental and computational challenge.

The authors present their findings and rationale very coherently, the methods employed are well justified, and overall, this study is of high caliber. So, my recommendation is to accept with minor revisions.

Minor revisions

1.) The ground truth for comparing your results is experimental data. However, one potential control is missing when comparing the relative performance of protein systems studied here. Precisely, experimental affinities were measured with different methods. These methods are noted in Figure 1—SPR, ITC, and NMR. For example, affinity measurements with ITC have an enthalpic dependence. Therefore, it could strengthen the results section, or discussion, to discuss what, if any, differences may arise from varied experimental methods. It may be worth noting this when comparing the performance of ABFE across all systems (results section for Figure 3).

2.) Without performing any quantitative analysis it looks to me that the data may include outliers in Figures 6a, 6d, 7b. Please check for outliers, and if they exist, briefly explain the origin of these data points, such as potential issues of convergence, and present regression analysis with outliers excluded.

3.) Regarding the systematic shift when comparing the ABFE values to experimental affinities, could you briefly explain the potential sources of this shift in your dataset? This is noted on pg. 17 at “For the most part ... this seems to represent a systematic shift in the free energy….” There appears to be a systematic shift in Figure 3 also.

4.) There is a few grammatical error on pg. 16: “These large sampling errors are reflect the charged nature....”

5.) The readability would improve without double negatives such as “The computational cost of ABFEs is not insignificant” on pg. 17.

Reviewer #4 (Remarks to the Author):

The manuscript describes calculations of the binding free energy for a larger number of fragments on four targets and addresses the question if such calculations would have been useful to take the relevant decisions in an optimization process. While retrospective analyses of this kind are difficult to assess, as the decisions have already been made (and possibly for different reasons), the manuscript shows what the current possibilities and challenges are. It seems that the binding free energies themselves are still off by some kcal/mol, but that a reasonable number of design decisions are supported by the calculations. The key message of this work seems to be that so-called absolute binding free energy calculations (as opposed to relative binding free energy calculations) can be used for fragments. This is in line with previous reports in this journal for complete compounds (ref 94 of the manuscript).

I would like to make the following comments:
1. The authors show that absolute binding free-energy calculations can be used for fragments, but do not really answer the question if these are more suited than relative binding free energies. One could even imagine setting up the relative calculations such that one fragment disappears fully, while another one appears, such that the active site is never completely empty. This could lead to a lower need to rearrange the active site. The authors could say a few more words about the merits and challenges of using absolute binding free energies.
2. I would recommend that the authors conclude their work with a clear recommendation. It seems that while the RMSE deviation from the experimental data is quite big, the actual decision making is mostly hampered by too large uncertainties? Should the field focus on improving the precision or on improving the accuracy?
3. The authors compare their approach with a cheaper MM/GBSA approach. They should say a bit more about the way they apply this method. I think they use a single trajectory approach, which may be suitable for relatively rigid fragments, but is usually not for larger compounds. They also do not seem to include an entropic contribution?
4. In the comparison to the MM/GBSA calculations it would be interesting to note if the same fragments lead to the largest deviations from experiment? Correlate the ABFE against the MM/GBSA data to see if the errors to experiment come from the force field (both approaches would correlate well), or from sampling issues (insufficient sampling of the difference between holo and apo proteins / lack of entropic contributions in MM/GBSA; no correlation between the two methods)
5. The authors find that the RMSE error is quite large for these fragments, but do not analyse in more detail where this discrepancy comes from. Is there an constant offset (MCL-1, Cyclophilin D) or a wrong slope (PWWP1, HSP90) ? A too steep slope is often the result of insufficient sampling, while an offset could rather point at a systematic error, e.g. in the force field. The authors may want to expand a bit on the sources of the errors?
6. I don’t understand what the authors intended with the sentence ‘to reproduce the work of Rocklin et al’. The cited work uses a correction scheme to correct for artefacts of charge changing calculations. What the authors do is a co-alchemical approach in which a counter ion is changed simultaneously with the ligand. Cite recent analyses of this approach, such as https://doi.org/10.1021/acs.jctc.8b00825 or https://doi.org/10.1021/acs.jctc.0c00719
7. The authors should probably cite ref 94 already on page 4.
8. The authors may want to cite similar retrospective analyses of free energy calculations and their agreement with design processes, e.g.: https://doi.org/10.1021/acs.jcim.0c00132

9. In the last paragraph of the conclusions, the authors mention methods to account for multiple binding modes of the fragments by either restraining them, or by enhancing the sampling. There are also approaches in which multiple binding modes are explicitly taken into account, see e.g. https://dx.doi.org/10.1016/j.bpj.2010.02.034

Response To Reviewers

Reviewer 1:

> One thing that I felt was missing from and could be added to the discussion is the influence of receptor holo-conformations on the results. The authors note the regular overprediction of ABFEP dG values compared to experiment clearly visible in Fig 3b-e. Since ABFEP calculations decouple ligands from a binding pocket, but do not give the binding pocket time to fully relax to its apo-state, the free energies would need to be corrected by this receptor strain contribution. In practice, this is difficult to do and not relevant for actual design, since even with ABFEP only the relative ranking of molecules matters. Therefore I believe it would be fair to adjust the computed results (e.g. with a constant offset that minimizes computed dG error compared to experiment, like it is done for RBFEP) for that and both present the relevant statistics for these dG results as well as discuss the amount of offset needed as an indication of receptor strain.*

We thank the reviewer for highlighting this important point. We have added further calculations for the Cyclophilin D and HSP90 systems, for which known *apo*-state crystals exist. We show that calculations of the ligands starting from *apo*-state crystals do not show a greater than error influence on the free energies (see revised Tables S5 and S12). Nevertheless, it is likely that such protein conformational changes do indeed have an impact on the overall free energy for these systems, however there are likely also other factors at play here. It is also likely that this may differ considerably between systems. Therefore, it is our view that it may not be correct to apply a set offset to our results based on the assumption that they all stem from the same sources of error.

> The authors note a limitation of their results, namely that their accurate scoring depends on knowing the right bound pose of the fragments. This is not realistic in a practical applications setting, but this concern could be eased by running a few ABFEP calculations with 'wrong' poses. Surely, many fragments could be flipped in the pocket or redocked into other parts of the receptor pocket. If ABFEP were able to pick out the 'correct' bound pose among these, a major limitation of the presented method would be removed.

We thank the reviewer for this interesting question. The small size and low affinity that fragments typically exhibit means that pose prediction of fragments is extremely challenging indeed. Nevertheless, we attempted to do a preliminary analysis of this by redocking two ligands from the PWWP1 set and evaluating their free energies using ABFEs (**Supplementary Text S6 and Table S16**). Whilst we were successful in identifying the lowest energy pose as the native pose for the larger of the two fragments, this was not the case of the for the smaller ligand. We conclude that further work would be required here to suitably assess the use of orientationally restrained ABFEs for this purpose, but it will also be useful to explore to what extent metrics like ligand-efficiency correlate with accuracy of pose-prediction.

> There seems to be a mangled sentence on page 11 "...decision occurs when in that set..."

We thank the reviewer for identifying this. The sentence has been re-written accordingly.

> I think giving RMSE values for MMGBSA is not a fair assessment of the method performance, MMGBSA is not usually expected to give quantitative scores in this fashion and such errors are always huge. To compute a meaningful RMSE, one would at least have to apply the same constant offset shifting to match experimental averages as suggested above or done in RBFEP.

The reviewer makes a fair point. We have thus removed the comparison of RMSEs between the results of the Nwat-MM/GBSA and ABFE methodologies.

Reviewer 2:

> 1) Main text, Page 9: It is mentioned that five independent replicas are simulated for each ABFE simulation using different orientational restraints. The selection of the orientational restraints is performed using MDRestraintsGenerator tool in the final 20 ns NPT equilibration simulation. The frame closest to the mean bond, angle and dihedral values of the chosen restraint over the 20 ns simulation is used as the starting point of the ABFE simulations. Based on the selection of the restraints it would be helpful if authors state whether they also use different starting points for each replica. If not, could the authors explain how they pick the rest of the orientational restraints that they are using in the ABFE calculations?

Here we start from the same initial structure for all replicas, but independently equilibrate each replica. Due to the rapidly diverging nature of MD simulations, the averaged restrained conformations obtained via MDRestraintsGenerator end up occupying different conformations between replicas. This is done intentionally in order to help us better capture the impact of conformational flexibility on the calculated free energy.

We have clarified the text in the manuscript's method section to address this query.

> 2) Main text, Page 12 and Page 18: In Figures 3 and 6, the experimental technique for each FBDD set is written in the x-axis except for the MCL-1 dataset. Could the authors add the experimental technique for this dataset as well?

Apologies for this oversight, the assay (FPA) has been added to both figures. We have also updated Figures 5 and 7 to match this style.

> 3) Main text, Page 17: The authors discuss a systematic shift in free energy of the Cyclophilin D dataset by approximately 2.5 kcal/mol. Could the authors provide a possible explanation for this shift, as they have done in the other datasets?

Thank you to the reviewer for raising this. This has been a source of frustration for us. Unfortunately, we have been unsuccessful in identifying the exact cause of this shift. We have explored a number of possibilities, but so far it does not appear to be related to conformation differences between the apo and holo states, nor does it seem linked to the presence of waters or ligand flexibility. Force field accuracy may play a role here, and testing different force fields is something which we aim to test in future work as this aspect would require a significant investment of compute and time beyond the scope of the current work. We have added relevant text to our discussion to highlight this.

> 4) Main text, Page 17: The authors claim that the 6 nM ligand 16, is estimated as within 0.3 kcal/mol of the 660 nM ligand 39. However, based on Table S10, we can observe that ligands 16 and 39 have similar experimental values (ΔGexp = -8.42 kcal/mol for ligand 16 and ΔGexp = -8.43 kcal/mol for ligand 39). In addition, the reported binding free energies based on ABFE calculations are not within 0.3 kcal/mol (ΔGABFE = -10.54 ± 0.48 kcal/mol for ligand 16 and ΔGABFE = -12.62 ± 0.47 kcal/mol for ligand 39). Could the authors kindly provide an explanation for this claim?

Thank you for spotting this. This was unfortunately a simple typo and should have been ligand 14 not ligand 16. We have updated the text accordingly.

> 5) Main text, Page 19: "We also show that ABFE calculations ………………….. offer comparable results to relative binding free energy methods". It would be nice if the authors can also add in Supplementary Information, a table containing an evaluation of the ligand elaboration steps of the MCL-1 ligand set for the RBFE calculations performed by Steinbrecher et al. In this way, they will be also to compare the predictive ability of relative binding free energy methods compared to ABFE calculations.

We thank the reviewer for this suggestion. An additional table outlining the elaboration decisions for the subset of MCL-1 ligands covered by Steinbrecher et al.'s work has been added in the supplementary information (**Table S10**). Additional text has been added in the main manuscript to describe this. We note that a direct comparison between the ABFE and RBFE results here is difficult as the FEP+ results did not provide errors between repeated calculations. However the relatively low cycle closure errors likely indicate that the elaboration decisions would be more clearly identified using FEP+ compared to the ABFE results.

> 6) Supporting Information, Page 5: In Table S2, ligand 14 is not present in the ligand elaboration steps of the PWWP1 ligand set. It would be helpful for the reader to point out why this ligand was included in the ABFE calculations since it is not present in the fragment optimization process.

Thank you for raising this. The synthesis of Ligand 14 is rationalised by Bottcher *et al* based upon the binding of a ligand which was not part of our dataset (ligand 7) as described here: "The quinoline-substituted derivative 14 (TR–FRET; IC50 = 3.9 μM) exhibited improved potency when compared to its pyridine analog 7 (TR–FRET; IC50 = 13 μM)". Analog 7 being a large peptide mimetic was not suitable for ABFE calculations and therefore was not included in our set. This led to ligand 14 not being included in the elaboration decisions.

We have added extra text to the manuscript to better explain this.

> 7) Supporting Information, Page 9: In elaboration decision 4, 18-24 perturbation yields better improvement in affinity for both experimental and computational techniques. Could the authors explain why they have placed "No" in the Right direction section?

We thank the reviewer for identifying this. In this particular case, we identify the free energy change between ligands 18 and 24 to be in the wrong direction based on the fact that ligand

24 should have a much higher affinity than ligands 25 and 26 which are involved in the same decision step. We have added extra context to the table's text in order to outline this decision.

> 8) Supporting Information, Page 12: Authors should add a negative sign to the acid groups in Figure S3. This will demonstrate the charged nature of the ligands.

Yes, we agree. Positive and negative signs to highlight charged groups have now been added to the ligand structures of PWWP1 and MCL-1.

> 9) Supporting Information, Page 13: In Table S7, ligand 1 is predicted to have a positive binding free energy. I believe that this is probably a typo (based on Figure 3 in the main text), but can the authors provide an explanation for this behaviour if this is not the case?

This is indeed a typo, thank you for highlighting this. This has now been fixed in the manuscript.

> 10) Supporting Information, Page 20: The authors provide an evaluation of the use of hydrogen mass repartitioning scheme (HMR) in GROMACS ABFE calculations, using both standard masses and HMR. It would be useful for the reader, if authors add a table with the predicted binding free energies for both schemes, using 5 and 20 replicas.

We thank the reviewer for this suggestion. An additional table (**Table S15**) has been added in the supplementary information showing the predicted free energies using both schemes.

> Typos:

- Main text, Page 8, Fig 2: Replace "ligand electrostatic" with electrostatics.

- Main text, Page 11: References for the 4 FBDD sets have been placed again as 89, 90, 91, 92.
- Supporting Information, Page 13: In Table S10, replace "HSP90" with "Cyclophilin D".

Thank you for highlighting these, appropriate changes have been made in the manuscript.

> In GROMACS a nonbonded exclusion bug that is described in detail in https://gitlab.com/gromacs/gromacs/-/issues/3403 is causing issues with FEP calculations. Essentially, excluded, perturbed pairs can end up in the pairlist at distances beyond rlist when the box is small due to a perturbed molecule ending up in the pairlist with its periodic image.

A documentation fix was written 1 month ago here: https://gitlab.com/gromacs/gromacs/-/commit/f54eaf30c9a626892d726b5085b9884780cbc6b0

Please address how you deal with this bug in your calculations and whether it affects your results. Also, please address this nonbonded exclusion bug in the manuscript, as researchers who want to perform ABFE calculations with GROMACS should be alerted about this bug in the software.

Thank you to the reviewer for raising this. The non-bonded exclusion bug highlighted in the above mentioned gitlab issue 3403 does not affect our results due to our use of a charge annihilation scheme rather decoupling the partial charges into gas phase. We have clarified this in the methods. The second issue highlighted, regarding the inadvertent crashes encountered when perturbing molecules inside of a small box also do not apply here. Our use of a 1.2 nm solvation distance from the solute and a 1 nm short range cut-off avoids this specific issue. Had either of these have affected our results the 2021 version of gromacs (which we employed for our ABFEs) would have led to deterministic software crashes, which were not observed here.

Reviewer 3:

> 1.) The ground truth for comparing your results is experimental data. However, one potential control is missing when comparing the relative performance of protein systems studied here. Precisely, experimental affinities were measured with different methods. These methods are noted in Figure 1—SPR, ITC, and NMR. For example, affinity measurements with ITC have an enthalpic dependence. Therefore, it could strengthen the results section, or discussion, to discuss what, if any, differences may arise from varied experimental methods. It may be worth noting this when comparing the performance of ABFE across all systems (results section for Figure 3).

The reviewer raises an important point. Indeed, our preference would have been to have constructed data sets with the same underlying experimental method, but this was not possible. We have added some text to the first part of the Methods section where we outline the choice of system for studies. With only 4 data sets, it is not really possible to delve into possible correlations between methods and overall performance at this stage.

> 2.) Without performing any quantitative analysis it looks to me that the data may include outliers in Figures 6a, 6d, 7b. Please check for outliers, and if they exist, briefly explain the origin of these data points, such as potential issues of convergence, and present regression analysis with outliers excluded.

We thank the reviewer for this suggestion. We have included a short analysis of outliers using the RANSAC algorithm in Supplementary information (**Text S5 and Figure S9**) and has been briefly referenced in the main text.

> 3.) Regarding the systematic shift when comparing the ABFE values to experimental affinities, could you briefly explain the potential sources of this shift in your dataset? This is noted on pg. 17 at "For the most part ... this seems to represent a systematic shift in the free energy…." There appears to be a systematic shift in Figure 3 also.

We thank the author for raising this. We have expanded the results and discussions in the manuscript to attempt to address the potential sources of these errors. As mentioned in response to Reviewer 1, this has been a source of frustration for us and thus far we have unable to identify the exact cause of this shift, but so far it does not appear to be related to conformation differences between the apo and holo states (see Table S12), nor does it seem linked to the presence of waters or ligand flexibility. Unfortunately, we have to conclude in the

discussion that there is insufficient evidence at this time for us to accurately tell what causes this poor accuracy. Future work using better sampling methods and/or higher quality force fields will aim to investigate these further.

> 4.) There is a few grammatical error on pg. 16: "These large sampling errors are reflect the charged nature...."

We thank the reviewer for highlighting this. The sentence has been fixed accordingly.

> 5.) The readability would improve without double negatives such as "The computational cost of ABFEs is not insignificant" on pg. 17.

We have gone through and removed such double negatives.

Reviewer 4:

> 1. The authors show that absolute binding free-energy calculations can be used for fragments, but do not really answer the question if these are more suited than relative binding free energies. One could even imagine setting up the relative calculations such that one fragment disappears fully, while another one appears, such that the active site is never completely empty. This could lead to a lower need to rearrange the active site. The authors could say a few more words about the merits and challenges of using absolute binding free energies.

This is indeed an interesting point and we thank the reviewer for highlighting it. We do note that works such as https://pubs.acs.org/doi/abs/10.1021/acs.jctc.0c01004 have shown that RBFE methods can be used for more complex fragment optimisations such as fragment linking. However, in cases such as what the reviewer proposes, i.e. making a ligand fully disappear whilst another re-appears as part of an RBFE, in our opinion this essentially amounts to doing two parallel ABFE calculations but adding the extra complexity of attempting to have a small substructure overlap. In cases such as those shown in the Cyclophilin D set, it is our opinion that ABFEs are easier to setup and have fewer intermediate steps than attempting to cover large perturbations via RBFEs, particularly given the additional advantage of getting an absolute measure of the binding free energy. That being said, to our knowledge, no complete comparison of the efficiency between the two methods have been made to date. Whilst we cannot carry out this evaluation as part of the work shown here, we hope that it may form the basis of future work.

A few additional sentences have been added to the discussion to cover this very interesting point.

> 2. I would recommend that the authors conclude their work with a clear recommendation. It seems that while the RMSE deviation from the experimental data is quite big, the actual decision making is mostly hampered by too large uncertainties? Should the field focus on improving the precision or on improving the accuracy?

We thank the reviewer for this point. Some additional sentences have been added to the discussion regarding this. Overall, it is difficult to make a clear recommendation to focus on either precision or accuracy. Indeed, in many cases optimizing one limitation will affect the other. For example, when improving sampling in order to improve precision, one can eventually encounter accuracy limitations (often force field based), which will hinder precision improvements. Thus, our recommendation here is that both precision and accuracy need to

be tackled at the same time in order to obtain meaningful improvements towards highly accurate ABFE calculations.

> 3. The authors compare their approach with a cheaper MM/GBSA approach. They should say a bit more about the way they apply this method. I think they use a single trajectory approach, which may be suitable for relatively rigid fragments, but is usually not for larger compounds. They also do not seem to include an entropic contribution?

We thank the reviewer for highlighting this. Additional details have been added to the methods section to clarify that this NWAT-MM/GBSA calculation uses a single trajectory approach and no entropy correction, as is the usual case for the NWAT methodology. We also noted in our discussion that further improvements by accounting more explicitly for entropy may lead to improved results in future works.

> 4. In the comparison to the MM/GBSA calculations it would be interesting to note if the same fragments lead to the largest deviations from experiment? Correlate the ABFE against the MM/GBSA data to see if the errors to experiment come from the force field (both approaches would correlate well), or from sampling issues (insufficient sampling of the difference between holo and apo proteins / lack of entropic contributions in MM/GBSA; no correlation between the two methods)

We thank the reviewer for raising this interesting analysis. Analysis of the correlation of signed errors between calculated free energies and experiment has been added to Supplementary information (**SI Text S4, Fig S7 and S8**). This analysis has also been referenced in the main text.

> 5. The authors find that the RMSE error is quite large for these fragments, but do not analyse in more detail where this discrepancy comes from. Is there an constant offset (MCL-1, Cyclophilin D) or a wrong slope (PWWP1, HSP90) ? A too steep slope is often the result of insufficient sampling, while an offset could rather point at a systematic error, e.g. in the force field. The authors may want to expand a bit on the sources of the errors?

We have expanded the results and discussions in the manuscript to attempt to address the potential sources of these errors. Unfortunately, additional simulations did not allow us to narrow down the specific causes of some of the larger errors seen. We conclude in the discussion that there is insufficient evidence at this time for us to accurately tell what causes this poor accuracy, but future work will aim to investigate these further.

> 6. I don't understand what the authors intended with the sentence 'to reproduce the work of Rocklin et al'. The cited work uses a correction scheme to correct for artefacts of charge changing calculations. What the authors do is a co-alchemical approach in which a counter ion is changed simultaneously with the ligand. Cite recent analyses of this approach, such as https://doi.org/10.1021/acs.jctc.8b00825 or https://doi.org/10.1021/acs.jctc.0c00719

We apologise for the confusion here. As stated under "Analysis" in our methods we do use the analytical correction as introduced by Rocklin et al. and not an alchemical ion. We have updated the above referenced text to clarify this. The point being made by this text is that the

fully coupled state has an excess charge equal to that of the ligand charge in order to match the work done by Rocklin et al. (rather than having the excess charge in the decoupled state).

> *7. The authors should probably cite ref 94 already on page 4.*

Thank you for raising this. We have added reference 94 on page 4 under works having shown highly accurate estimates of binding free energies via ABFE.

> *8. The authors may want to cite similar retrospective analyses of free energy calculations and their agreement with design processes, e.g.: https://doi.org/10.1021/acs.jcim.0c00132*

Thanks to the reviewer for highlighting this. We have added that to the Discussion

> *9. In the last paragraph of the conclusions, the authors mention methods to account for multiple binding modes of the fragments by either restraining them, or by enhancing the sampling. There are also approaches in which multiple binding modes are explicitly taken into account, see e.g. https://dx.doi.org/10.1016/j.bpj.2010.02.034*

We thank the reviewer for this. We have now included this type of approach in the Discussion.

REVIEWERS' COMMENTS:

Reviewer #1 (Remarks to the Author):

The authors have reworked their manuscript to address all points I raised. I think it can be published as is now.

Reviewer #4 (Remarks to the Author):

The authors have addressed my comments satisfactorily. Several typo's appear in the newly added texts, but this can be corrected at the proofs stage.

In response to the suggestion I made in my first point ("One could even imagine setting up the relative calculations such that one fragment disappears fully, while another one appears, such that the active site is never completely empty.") the authors respond that this "this essentially amounts to doing two parallel ABFE calculations but adding the extra complexity of attempting to have a small substructure overlap."
a) There is no need for a small substructure overlap, it could consist of a single distance restraint between two atoms of the two fragments. Or as there are restraints between the fragments and the protein already, this may not be necessary at all.
b) I do not quite agree with the opinion of the authors that it amounts to doing two parallel ABFE calculations, because it removes the need to sample two, typically slow, processes: 1) resolvation of the protein active site when when a fragment disappears, and 2) any conformational changes between the holo and apo protein structures (which are typically larger than the conformational differences between two holo structures.
I do not think these aspects need to be addressed in this manuscript, but I hope that the authors will consider my thoughts in their future work.
